# Rice biofortification: breeding and genomic approaches for genetic enhancement of grain zinc and iron contents

**DOI:** 10.3389/fpls.2023.1138408

**Published:** 2023-06-02

**Authors:** P. Senguttuvel, Padmavathi G, Jasmine C, Sanjeeva Rao D, Neeraja CN, Jaldhani V, Beulah P, Gobinath R, Aravind Kumar J, Sai Prasad SV, Subba Rao LV, Hariprasad AS, Sruthi K, Shivani D, Sundaram RM, Mahalingam Govindaraj

**Affiliations:** ^1^ Crop Improvement Section, ICAR - Indian Institute of Rice Research (ICAR - IIRR), Hyderabad, India; ^2^ Genetics and Plant Breeding, Professor Jayashankar Telangana State Agricultural University (PJTSAU), Hyderabad, India; ^3^ HarvestPlus, Alliance of Bioversity International and the International Center for Tropical Agriculture (CIAT), Cali, Colombia

**Keywords:** rice, biofortification, iron, zinc, breeding, genomics

## Abstract

Rice is a highly consumed staple cereal cultivated predominantly in Asian countries, which share 90% of global rice production. Rice is a primary calorie provider for more than 3.5 billion people across the world. Preference and consumption of polished rice have increased manifold, which resulted in the loss of inherent nutrition. The prevalence of micronutrient deficiencies (Zn and Fe) are major human health challenges in the 21^st^ century. Biofortification of staples is a sustainable approach to alleviating malnutrition. Globally, significant progress has been made in rice for enhancing grain Zn, Fe, and protein. To date, 37 biofortified Fe, Zn, Protein and Provitamin A rich rice varieties are available for commercial cultivation (16 from India and 21 from the rest of the world; Fe > 10 mg/kg, Zn > 24 mg/kg, protein > 10% in polished rice as India target while Zn > 28 mg/kg in polished rice as international target). However, understanding the micronutrient genetics, mechanisms of uptake, translocation, and bioavailability are the prime areas that need to be strengthened. The successful development of these lines through integrated-genomic technologies can accelerate deployment and scaling in future breeding programs to address the key challenges of malnutrition and hidden hunger.

## 1 Introduction

**Figure 1:**
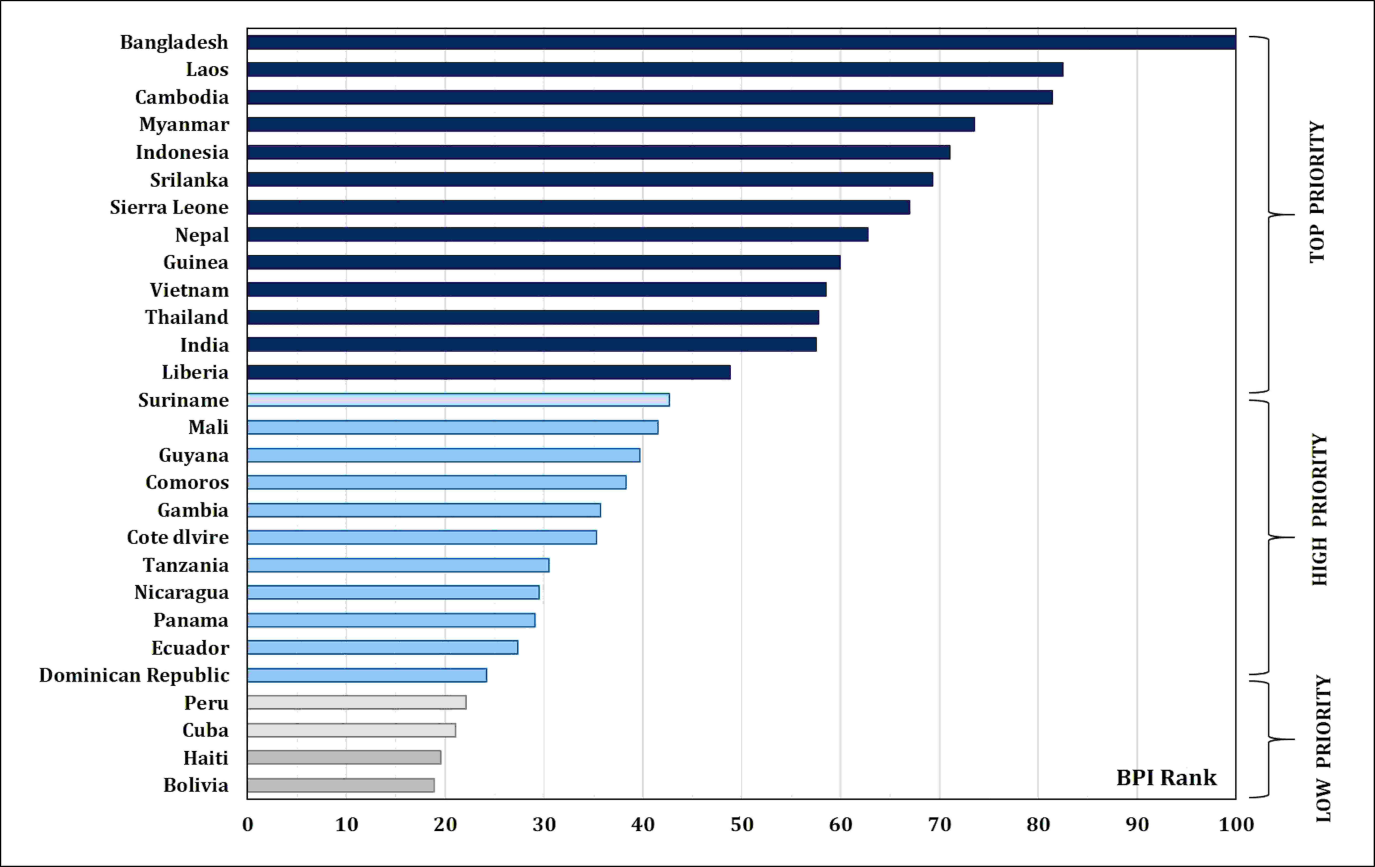
Biofortification priority index (BPI) global rankings and priority levels for rice-consuming countries in Asia, Africa, and Latin America and the Caribbean (LAC).

Micronutrient malnutrition is also known as “hidden hunger”. It has become one of the major public health concerns across the world. In addition, micronutrient deficiencies in poor households are also responsible for lower productivity and lower income. Globally, 828 million people were facing hunger in 2021. Hence, the need for a healthy diet has been recognized and included in 12 of the 17 Sustainable Development Goals set by the United Nations (FAO et al., 2022). The State of Food Security and Nutrition in the World has to feed the projected 10 billion people in 2050 through sustainable agricultural production and consumption practices. According to World Health Organization reports, iron (Fe) and zinc (Zn) are among the most important micronutrients; however, their availability is inferior in most diets (https://www.who.int/). Nearly 31% of the world’s population suffers from Zn deficiency. Consequently, the annual child mortality is more than 400 million. Up to 80% of the world’s population suffers from insufficient Fe, and 30% of them are anemic due to long-term Fe deficiency. Annually, Fe deficiency causes 0.8 million deaths (Midya et al., 2021). Anemia is also prevalent in 23% and 52% of the developed and developing countries, respectively, hence the necessity for global investment in health interventions (WHO, 2015). India has the highest number (61.7 million of the 162 million in the globe) of stunted children under the age of 5 years (NFHS-5, 2020). In order to achieve better health in the ever-growing population, it is imperative to collectively address malnutrition gradually and sustainably. In 2020, the Food Safety and Standards Authority of India (FSSAI) recommended a dietary allowance of Fe (17, 21, and 35 mg/day for men, women, and pregnant women, respectively) and Zn (12, 10, and 12 mg/day for men, women, and pregnant women, respectively) (https://www.fssai.gov.in/RDA). For half of the world’s population, rice serves as the primary staple food and accounts for more than 50% of daily calorie intake (https://education.nationalgeographic.org/resource/food-staple). Zn intake through zinc-biofortified rice consumption was estimated at 2.9 g/day per person (this was based on rice consumption of 220 g/day per person) (Sanjeeva Rao et al., 2020). HarvestPlus program (of the Consultative Group on International Agricultural Research (CGIAR) that leads the global biofortification initiatives), through systematic expert consultations, set a breeding target of 13 μg/g of Fe and 28 μg/g of Zn in polished biofortified rice to reach 30% of the estimated average requirement (EAR) of daily consumption (Trijatmiko et al., 2016; Matres et al., 2021) (**Table 1**). HarvestPlus prepared the biofortification priority index (BPI), which is a global index for most staple crops that clearly shows the need for rice Zn biofortification in Asia (Bangladesh, Laos, Cambodia, Myanmar, Indonesia, Sri Lanka, Nepal, Vietnam, Thailand, and India) and Africa (Sierra Leone, Guinea, Madagascar, Guinea-Bissau, Liberia, Mali, Comoros, Gambia, Cote d’Ivoire, and Tanzania) (HarvestPlus, 2020) (**Figure 1**). Considering the significance of rice biofortification with Zn as a primary trait and Fe as an associated trait for better human health, the present review discusses the dynamics of Zn and Fe distribution and their accumulation in rice grain, genetics and breeding strategies in dissecting the genetic loci governing micronutrient content in rice, and recent genomic, transgenic, and advanced biotechnological approaches for the long-term integration of biofortification in rice.

**Table 1:** Daily requirement of micronutrient (Fe, Zn, and protein) content for human nutrition.

Nutrient	Baseline	Daily requirements		Reported bioavailability range
		Women	Men	
**Iron (Fe)**	45 mg/day (for infants 20 mg/day)	8–18 mg/day (pregnant women 22–27 mg/day)	8 mg/day	1.08 ± 0.02 mg/100 g in rice
**Zinc (Zn)**	35–40 mg/day (for infants and children 4–25 mg/day)	6.5–8 mg/day (pregnant women 8–11 mg/day)	12–14 mg/day	1.32 ± 0.03 mg/100 g in rice
**Protein**	–	37–57 g/day (pregnant women 47–60 g/day)	52–64 g/day	

## 2 Role of zinc and iron in human health

Zinc is the most essential micronutrient in human health required for enzymatic activity, tissue growth and development, protection against infectious diseases, taste acuity, bone mineralization, proper thyroid function, blood clotting, cognitive function, fetal growth, and immunity (Bhowmik et al., 2010; Prasad, 2013; Gammoh and Rink, 2017; Calayugan et al., 2021). Zinc deficiency ranked as the fifth leading risk factor for diseases in the developing world (Maret and Sandstead, 2006). Approximately one-third of the world’s population is suffering from zinc deficiency (Brown et al., 2004) and is one of the major causes of child mortality in more than 178 countries (Black et al., 2008). Zn deficiency in grown children and adults may cause delayed growth, dwarfism, delayed sexual development, impaired sense of taste, poor appetite, and mental lethargy (Stein et al., 2007). A higher amount of Zn is required for infants, children, adolescents, and pregnant and lactating women, who are at higher risk. Stunting is widely used as an indirect (proxy) indicator of Zn deficiency since proof of concept for zinc in the human body is yet to be identified. Zinc is one possible factor in the overall equation of linear growth.

In contrast to Zn, Fe is an abundant element on earth and is an essential element for almost all living organisms for metabolic processes, like synthesis of oxygen transport proteins (hemoglobin and myoglobin) and electron transfer in oxidation–reduction pathways. Iron deficiency can exist with or without anemia with functional deficits like reduced learning ability and delayed physical and mental growth (Bouis et al., 2003). Iron deficiency during pregnancy affects adversely both the mother and infant, like increased risk of sepsis, maternal mortality, prenatal mortality, and low birth weight (Schmidt et al., 2014).

## 3 Mechanism of iron and zinc translocation in rice

**Figure 2:**
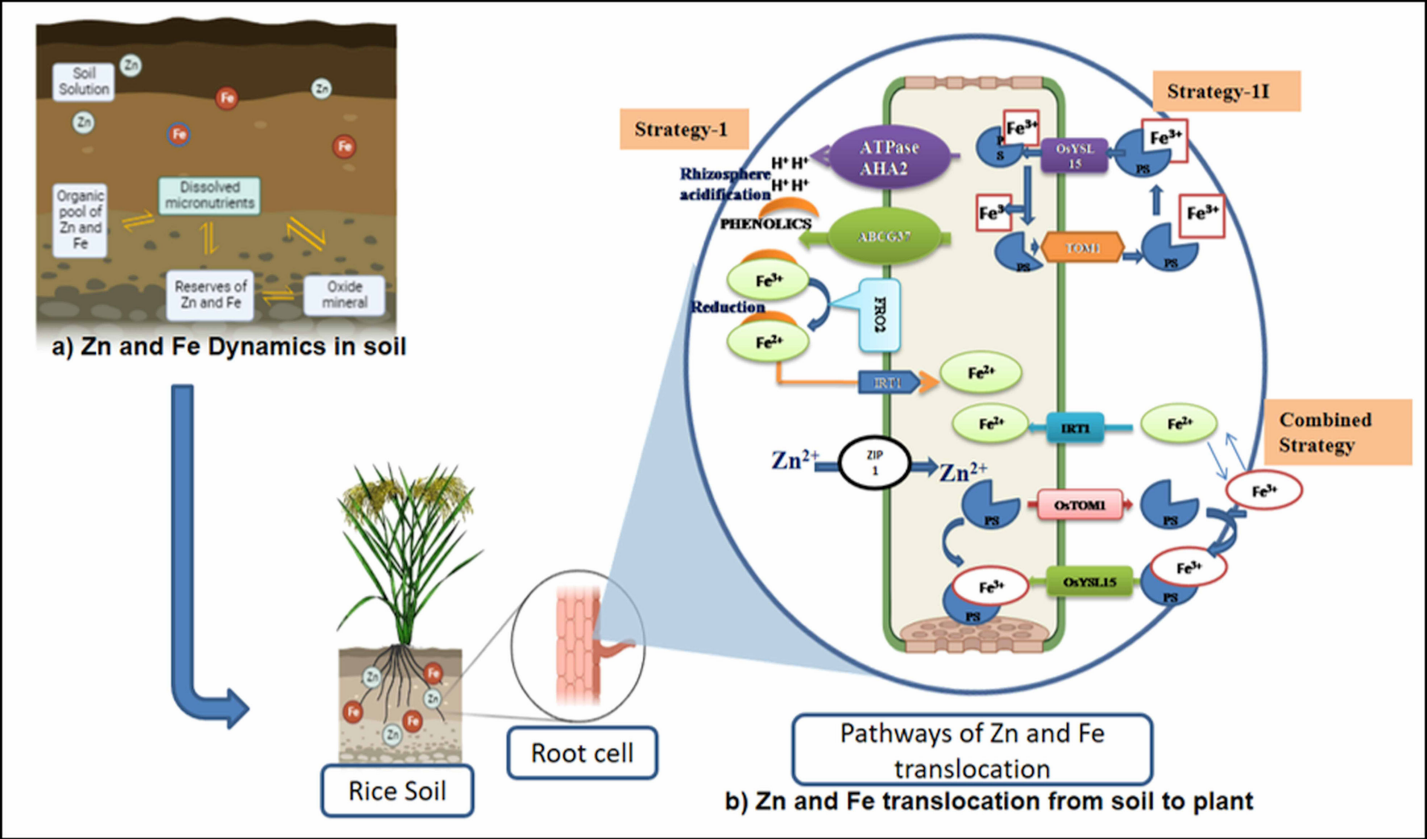
Soil strategies of micronutrient availability in crops. **(A)** Zn and Fe dynamics in soil. **(B)** Zn and Fe translocation from soil to plant. *OsYSL15*, an iron-regulated iron (III)-deoxymugineic acid transporter; *OsTOM1*, TRANSPORTER OF MUGINEIC ACID 1; IRT-1, Iron-Regulated Transporter 1; ZIP1, ZRT, IRT-like protein; FRO2 gene, Ferric Chelate Reductase gene; AHA2, *Arabidopsis* plasma membrane H^+^-ATPase 2 gene; ABCG3, The ATP-binding cassette (ABC) transporter.

The availability of micronutrients in the soil is largely influenced by various soil factors (soil pH, redox potential, organic matter, and cation exchange capacity), associated factors such as mineralization (ionization and complex formation), and uptake transporters. Iron is typically found in soil in two oxidative states, ferric (Fe^3+^) and ferrous (Fe^2+^), while zinc is present as Zn^2+^. Soil pH plays a major role in Fe and Zn availability (**Figure 2**). Soil pH is the highly influencing factor in nutrient availability for plant growth and predominantly regulates the availability of Zn and Fe in the soil. An equation was developed using various thermodynamic parameters and derived as follows: (Zn^2+^) = 105.8 × (H^+^)^2^ and (Fe^3+^) = 102.7 × (H^+^)^2^. The equation exhibits that the activities of cations are inversely related to pH. In general, activities and availability of divalent (Zn^2+^ and Fe^2+^) and trivalent (Fe^3+^) micronutrient cations decrease to the tune of 100- and 1,000-fold with a unit change in pH. For example, if the pH ranges from 5.5 to 7.0, an increase of one pH unit causes a 30–45-fold decrease in aqueous Zn^2+^ content and a 100–1,000-fold decrease in aqueous Fe^2+^ and Fe^3+^ contents (He et al., 2005; Rattan et al., 2015). Two main strategies were employed—strategy I (reduction process) and strategy II (chelation process)—in plants for the uptake of Fe from the rhizosphere (**Figure 2**). Reduction-based strategy occurs in non-grasses, *viz*., in dicots (legumes) and non-graminaceous monocots (Römheld and Marschner, 1986), and involves acidification of rhizosphere (protons are exuded to the rhizosphere) by the plasma membrane through the release of *H^+^ ATPase* from *AHA2.* The released proton acidifies the soil and increases the solubility of Fe^3+^, which is chelated by phenolic compounds (coumarin family) and exported by *ABCG37 transporter*. Chelated Fe^3+^ is reduced to Fe^2+^ by *Ferric Reduction Oxidase-2* (*FRO_2_*) in the plasma membrane. Fe^2+^ is transported by Iron Regulated Transporter 1 (IRT_1_) into root epidermal cells. Chelation strategy (so-called strategy II) occurs in grasses, especially in all major cereal crops including rice (López-Arredondo et al., 2013), which involves phytosiderophore release into the rhizosphere by superfamily transporters (*OsTOM1*/*OsZIFL4*), which chelates Fe^3+^ and forms complex (Fe^3+^–PS). Though rice adopts strategy II as the primary mechanism, it also exhibits strategy I mechanism partially or a combination of both strategies (Krohling et al., 2016; Swamy et al., 2021), depending on their growth condition and environment, which might have evolved before the domestication of cultivated rice (Wairich et al., 2019). In a low Fe-deficient soil condition, combined reactions like the exclusion of proton by the *AHA2H^+^*–*ATPase* and the formation of Fe(III)-DMA complex predominantly take place in the rice roots. It is further activated by *deoxymugineic acid efflux transporter* (*TOM1*), the Fe^3+^–deoxymugineic acid transporter (*OsYSL15*), and the Fe^2+^ uptake system through mono group Fe^2+^ transporters (*OsIRT1* and *OsIRT2*) expressed in rice roots to overcome the Fe deficiency-induced chlorosis (Ishimaru et al., 2006; Cheng et al., 2007; Krohling et al., 2016). These series of actions take place in a coordinated way to respond to Fe deficiency.

### 3.1 Availability of Fe and Zn in aerobic vis-à-vis flooded conditions

Zn and Fe bioavailability is a function of both soil factors and plant traits that can be altered by water management, particularly in the biological process of mycorrhizal inoculation and the release of organic compounds from the root to the rhizosphere (Gao et al., 2002). Because of the reduced state of soil (excess moisture content) and higher heat capacity, the dry direct seeded aerobic rice suffers from nutrient deficiency, as nutrient transport to soil is affected via mass flow and diffusion process (Midya et al., 2021). The excretion of organic acids or phytosiderophores capable of increasing the uptake of Zn in the rhizosphere acts as a potential mechanism for reducing Zn deficiency in lowland rice. When rice is grown in alkaline soil (anaerobic environment), microbial decomposition of organic matter results in the production of CO_2_-developed carbonic acid, which dissociates into H^+^ and bicarbonate (HCO_3_) followed by a reduction in the soil pH. In a similar way, roots are also involved in the extrusion of H^+^, while uptake of NH^4+^-N from the soil is followed by a consequent decrease in rhizosphere pH, and subsequently, an increase in Zn bioavailability for plants (Kirk and Bajita, 1995; Rengel, 2015) in a reverse shift to aerobic environment cultivation increases the pH due to release of OH^−^ by taking up of NO_3_-N, which simultaneously decrease the Zn and Fe availability by seizing them as hydroxides. In the case of aerobic rice cultivation, the shift in the dominant form of N uptake from NH_4_^+^ to NO_3_^−^ is expected, which leads to the exudation of OH^−^ into the rhizosphere resulting in an increase in rhizosphere pH with a subsequent reduction in Zn availability. The shift in rhizosphere pH as a result of changing N dynamics may be the cause of the reduced Zn uptake observed in rice grown in aerobic fields compared to flooded fields on calcareous soil (Gao et al., 2006).

Since flooding can cause the soil pH to increase in acid soils and decrease in sodic and calcareous soils, when aerobic soil is submerged, the pH decreases for the first few days and increases asymptotically to a stable value of 6.7 to 7.2 (Ponnamperuma, 1972). Zn^2+^ binds tightly to soil elements by forming zinc hydroxide [Zn(OH)_2_] and plant cell wall sections at high pH. Therefore, soil pH has a major effect on Zn acquisition and absorption from the rhizosphere by roots. Under aerobic conditions, the redox potential rises to the positive side, resulting in the formation of Fe and Mn oxides, onto which Zn could be adsorbed (Gao et al., 2002; Kirk, 2004; Bunquin et al., 2017). The switch to aerobic cultivation can result in an increase in the number and diversity of Fe-oxidizing/reducing bacteria (Chen et al., 2008) affecting Zn content and speciation in the soil solution. Reduced Zn content in plant tissue, Zn absorption, and Zn harvest index suggested lower Zn bioavailability in aerobic rice cultivation systems compared to flooded rice cultivation systems (Gao et al., 2006). Fe in acidic soils is ionized as Fe^2+^/Fe^3+^, while in aerobic alkaline soils, Fe is immobilized as Fe(OH)_3_. Fe-deficiency chlorosis in crops is most severe on calcareous soils due to the low availability of Fe in the presence of oxygen, especially at moderate and high pH (Graham and Stangoulis, 2003). In general, rice cultivated in an aerobic environment does not produce a significant amount of phytosiderophores, H^+^ (no change in pH of soil solution), and Fe^3+^ reductase activity to compensate for Fe under deficiency. These findings could help improve our knowledge of Fe-deficiency chlorosis in aerobic rice production. The low soil pH and phosphorus content were found to influence Fe content in grains, while soil Zn availability and electrical conductivity influenced Zn content in rice grains (Pandian et al., 2011). In aerobic situations, under Fe deficiency, it was confirmed that lower amounts of phytosiderophores are released from aerobic rice and reduced ferric ion reducing capacity, and pH did not decrease significantly unlike in flooded rice (Midya et al., 2021).

### 3.2 Fe accumulation in aleurone layer and Zn distribution

**Figure 3:**
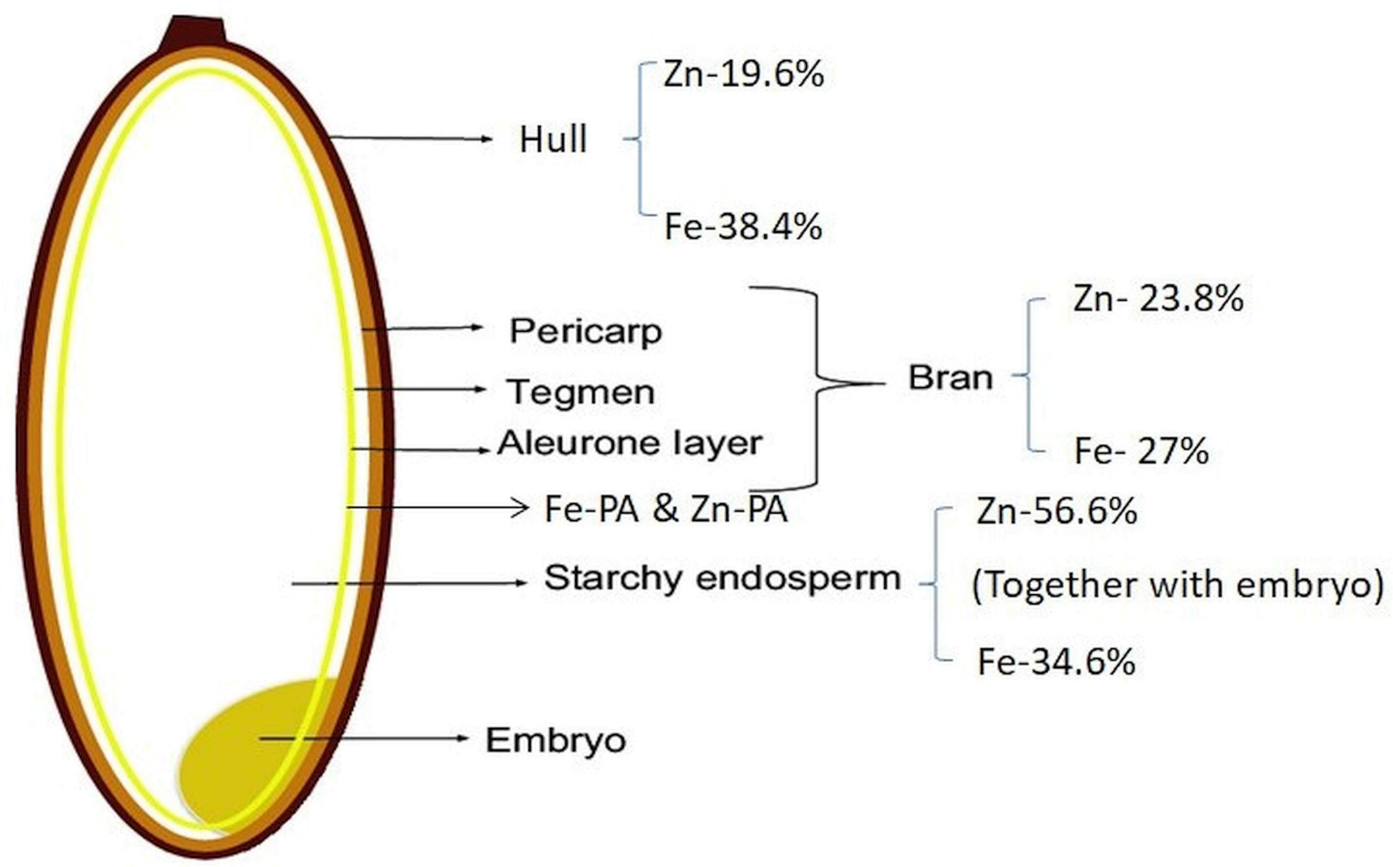
Percent distribution of iron (Fe) and zinc (Zn) in different parts of rice grain. Fe-PA, iron–phytic acid complex; Zn-PA, zinc–phytic acid complex.

Phosphorus translocated from source organs is converted to inositol hexaphosphate (InsP6) (phytic acid PA]) and accumulates most abundantly in the aleurone layer (Iwai et al., 2012). InsP6 has a high cation-binding affinity toward metal cations like calcium, potassium, and iron, thus, forming phytates, the salts of InsP6, whereas zinc loosely bound to InsP6 as not only phytate but also other storage forms (Iwai et al., 2012). In cereal grains, phytates are mainly deposited in the aleurone layer, whereas in legume seeds, phytates are accumulated in cotyledons and embryo axes. Phytic acid (InsP6) acts as a storage compound of phosphorus in seeds and Zn is loosely bound to InsP6. The InsP6 content in the outer parts of seeds rapidly increases during seed development, and the phosphate contents of both the outer and inner parts of seeds remain low (Yoshida et al., 1999). Fe is primarily bound to phytic acid, while Zn is bound to proteins, indicating that Fe and Zn have different speciation with different contents (**Figure 3**) in cereal grain tissues (Persson et al., 2009). The iron-chelate transporter of rice, *OsYSL9*, is a novel transporter for Fe(II)-nicotianamine and Fe(III)-deoxymugineic acid that is responsible for internal iron transport, especially from endosperm to embryo in developing seeds (Senoura et al., 2017). Based on the experimental results of Iwai et al. (2012) on dynamic changes in the distribution pattern of minerals during seed development of Kitaake and Nipponbare, at 10 days after flowering, Zn accumulated more around aleurone layer, later decreased, and moved into the inner endosperm through vascular bundles and translocated. Fe is immediately captured by phytic acid, which is a strong Fe chelator in the aleurone layer and prevents its movement to other parts of the seed. Micronutrients accumulate at five to six times higher concentrations in the storage vacuole of the embryo than in the endosperm (Saenchai et al., 2012). Two homologous genes—*OsVIT1* (highly expressed in flag leaf blade) and *OsVIT2* (highly expressed in flag leaf blade and sheath)—were involved in iron translocation between source and sink organs (Zhang et al., 2012). However, Che et al. (2021) identified that the enhanced Fe accumulation in polished rice grain is due to results from decreased vacuolar sequestration of iron, predominantly in the nodes. To improve iron and zinc concentrations in rice grains, transgenic rice plants co-expressing *NAS* with other genes are being generated by Wairich et al. (2022). Iron concentration increased over sixfold in rice endosperm when co-expressing *Ferritin* gene from *Phaseolus vulgaris* (*PvFer*) and *Arabidopsis thaliana NAS gene* (*AtNAS1*) (Wirth et al., 2009). Co-expression of *OsNAS1* and *barley nicotianamine aminotransferase* (*HvNAATb*) genes under maize ubiquitin promoter increased iron and zinc contents in the endosperm (Banakar et al., 2017; Díaz-Benito et al., 2018).

## 4 Breeding approaches for improving zinc and iron contents in rice

### 4.1 Genetic variation for grain Zn and Fe contents

Plant breeders rely on selection based on additive effects, non-additive genetic effects, recovery of transgressive segregants, and hybrid generation through heterosis breeding. Assessing micronutrient diversity and identification of potential donor parents by distance in the available but adaptable germplasm, popular rice varieties, or advanced breeding lines will help in broadening the genetic base of nutrient-dense biofortified rice pipelines through the pre-breeding programs. Most of the key traits in the combined germplasm lines (landraces, advanced breeding lines, and varieties) are described in **Table 2**. These levels are subject to validation with available HarvestPlus protocols (monitoring sample contamination) and laboratory standards for further use in the breeding program. The initial screening of rice germplasm for grain Fe and Zn indicated the existence of significant genetic variation for grain Fe and Zn by many research studies (Gregory and Fergus, 2017; Garcia-Oliveira et al., 2018). Wang et al. (1997) reported an average Zn content of 3.34 mg/100 g, which varied between 0.79 to 5.89 mg/100 g in 57 rice genotypes. Graham et al. (1999) reported grain Zn and Fe contents from 15.3 to 58.4 and 6.3 to 24.4 mg/kg, respectively, in 100 rice genotypes. Landraces were found to be superior for micronutrients especially Zn and Fe in comparison to popular varieties/adaptable genotypes (Gayacharan et al., 2018). Similarly, Vanlalsanga et al. (2019) reported high Zn content (75.8 mg/kg) in a landrace Badalsali among the other rice cultivars. From the review of several studies, it has been observed that the highest variation for Zn content in brown rice was 6.2 to 71.6 mg/kg (Anuradha et al., 2012); in the case of polished rice, the range was from 4.8 to 40.9 mg/kg (Sanjeeva Rao et al., 2020). Fe content in brown rice ranged from 6.9 to 67.3 mg/kg (Maganti et al., 2020 and Anuradha et al., 2012); in the case of polished rice, the range was from 0.8 to 36.45 mg/kg (Bollinedi et al., 2020 and Raza et al., 2019). It is reported that brown rice has higher Zn and Fe contents than polished rice (Raza et al., 2019 Sanjeeva Rao et al., 2020). The emphasis should be given more in pre-breeding for an increase of Zn and Fe contents in the polished rice, as the % loss during polishing depends on the degree and duration of polishing as well as location and variety.

**Table 2:** Promising donors identified for Fe and Zn in rice.

Nutritional element	Landraces	Advanced breeding lines/varieties	Micronutrient content (ppm)	Reference
**Iron (Fe)**	Zuchem and Jalmagna		20.2–22.4	Gregorio et al., 2000
	Taraori Basmati	HKR 95-157, Palman 579, HKR 95-130	207.5–441.5 (in dehusked rice)	Brar et al., 2011
	Kalabath, Norungan, Noothipattu and Pitchavari	Pusa Basmati 1 and White ponni	20.7–39.19	Anandan et al., 2011
	Chittimuthyalu		30.1–35.0 (in brown rice)	Sanjeeva Rao et al., 2020
	Nootripathu (RG192), CHIR8 (RG8), RG69 (Bindli), RG75		13.3–16.7	Nachimuthu et al., 2014
	Kalanamak and Chittimutyalu	Kanchana, Karjat 4, Udayagiri, Jyothi, VRM 7, MettaTriveni, Varsha, MSE 9	19.8–34.4 (in brown rice)	Ravindra Babu, 2013
	Swetonunia		34.8	Roy and Sharma, 2014
	Fazu, Kapongia, Ajaya, Phougak, Phourenamubi, Mezamew, Badalsali and Deku		86.96–215.62	Vanlalsanga et al., 2019
	Aghonibora and Profulla	IR-36, NDR6279	31.2–45.1	Jagadeesh et al., 2013
**Zinc (Zn)**	Jalmagna, Zuchem, XuaBueNue, Madhukar	IR64	23.2–34.2	Gregorio et al., 2000
	Taraori Basmati	Pusa 1460, TNG 67 and BG 951	34.6–39.4 (in dehusked rice)	Brar et al., 2011
	Subhadra, Chittimuthyalu, Kalanamak and Taroari Basmati,	ARB-55, ARB182, Sabita, 233, BPT5204/Chittimuthyalu, BPT5204/Sheshi, Kasturi	25.1–30.0 (in brown rice)	Sanjeeva Rao et al., 2020
	Kalabath, Pusa Basmati 1, Norungan, Noothipattu, Pitchavari and Black navara	Jalmanga, White ponni	26.8–38.6	Anandan et al., 2011
	vadakathi samba (RG187) and RG130 (Honduras)	RG149 (RH2-SM-2-23), RPHP 90 and RPHP 163	16.4–32.4	Nachimuthu et al., 2014
	Chittimutyalu and Ranbir Basmati	Poornima, ADT 43, Type 3, Udayagiri, Ratna, Jyothi, Pant Sugandh 17, Kesari	30.1–32.7 (in brown rice)	Ravindra Babu, 2013
	Nepali Kalam, Govindobhog, Begunbeej, Nepali Kalam and Gobindobhog		16.15–195.3	Roy and Sharma, 2014
		IR14M126, IR15M1319, IR15M1284, IR93337:37-B-15-15-22-1RGA-2RGA-1-B	19.65–23	Umar Farooq et al., 2019
	Badalsali, Taothabi, Heibo, Lumre, Mulong, Vak and Amoankodhan	RCM10 and RCM 13	51.4–75.8	Vanlalsanga et al., 2019
	Vasumathi, Thanu and Aghonibora		43–55	Jagadeesh et al., 2013

Higher variability of grain nutrients like Fe and Zn was observed in colored rice, i.e., brown and red than in white rice genotypes. Red rice has twofold to threefold higher Zn and Fe contents than white rice (Ramaiah and Rao, 1953). Graham et al. (2001) indicated that high Fe and high Zn contents are linked to aromatic varieties such as Jasmine and Basmati. The Fe and Zn contents in brown rice of aromatic fine-grain accessions were higher than in coarse-grain accessions (Raza et al., 2020).

### 4.2 Genetics of Zn and Fe contents

Understanding the trait’s inheritance and its heritability patterns are keys to devising an appropriate breeding methodology. The duplicate epistatic interactions that control the expression of grain Fe and Zn contents can be exploited through recombination and heterosis breeding coupled with selection in later generations, which could be an effective approach for increasing both grain Fe and Zn contents in rice (Kumar et al., 2020). The characterization of a wide range of germplasm collections unveiled significant variation for grain Zn content with moderate-to-high heritability and genetic advance (GA). The genetic variability and heritability studies of grain Zn and Fe contents from 2012 to 2022 showed moderate-to-high broad sense heritability (40.6% to 99.8%) for grain Zn content, low-to-high broad sense heritability for Fe (0.22% to 99.7%), and low-to-high genotypic coefficient variation (GCV) (7.6% to 45.1%) and moderate-to-high phenotypic coefficient variation (PCV) (11.3% to 45.3%). The slight variation between GCV and PCV values indicates the minor influence of environment and edaphic factors on the expression of these traits (Gregorio, 2002). Some studies suggest that single-plant selection is the best method for improving grain Zn content, as the heritability of the seed genetic effect and high levels of narrow-sense heritability are influencing factors (Swamy et al., 2016).

The gene action of grain Fe and Zn contents is non-additive (Kirubha et al., 2019; Lingaiah et al., 2021). The heterotic hybrids evaluated under direct seeded aerobic conditions showed higher micronutrient content coupled with grain yield than better parents, implying dominance gene action and also complementary non-allelic gene interaction action (Anusha et al., 2021). The combining ability studies showed that the additive genetic effects were more crucial for Zn content and considerably influenced by genotype and environment (G × E) interactions (Sharifi, 2013). For Zn content, the negative correlation between grain yield and micronutrient levels has been more significant, and the preponderance of site-specific soil zinc levels manifests as G × E × M interaction and ranking of high Zn content entries. Apparently, selecting genotypes that respond positively to increased soil Zn content could be a beneficial strategy in areas, where soil Zn levels are high (Naik et al., 2020). Whether the micronutrient traits have a preponderance of non-additive as well as both additive and non-additive genetic actions could be exploited through recombination breeding and can be improved by exercising selection pressure in later generations on these characters; if it is governing through additive genetic action, selection at early generation is effective (Sharma et al., 2021). The brown and red rice genotypes have high grain Zn and Fe contents, and the heterosis for these traits along with grain yield was reported (Rathna Priya et al., 2019). Forty-eight rice hybrids evaluated for micronutrient content, two hybrids for grain Fe content, and 14 hybrids for grain Zn content had positive significant heterosis over the standard variety, Chittimuthyalu (Nagesh et al., 2012). Introgressed breeding projects also have a positive impact on developing high rice varieties. Paul et al. (2014) successfully interbred IR68144 rice having 2.54-fold more iron and 1.54-fold more zinc in milled rice grain. Diverse *Oryza nivara* accessions, IRGC81848 and IRGC81832, were identified, which had twofold to threefold higher Zn and Fe contents when compared to the recipient parent Swarna (Swamy et al., 2018). However, breeding efforts have not advanced much for the development of Fe-rich polished rice varieties through conventional methods because of limited variation in polished rice (Bashir et al., 2013; Slamet-Loedin et al., 2015). As most of the Fe concentrated between one and five aleurone layers of different germplasm, the high Fe in brown rice can be due to the thickness of bran layers, and therefore, low Fe concentration was observed in polished rice (Slamet-Loedin et al., 2015).

### 4.3 Trait correlation and biofortification implication

Control of the homeostasis of Fe and Zn in rice is a challenging task, as it is governed by complex genetic and metabolic networks; hence, the development of biofortified rice is challenging. Internal mobilization patterns of these micronutrients can be influenced by variations in the use efficiency of Fe and Zn, variability in their concentration level in the grain, and genotype-dependent source–sink relations, which make it difficult for the selection of adequate genetic backgrounds to enhance Fe and Zn accumulation in the grain. Therefore, the knowledge of the genetic correlation of various traits with micronutrients plays a key role in developing biofortified varieties by a selection of more easily measured and highly correlated traits.

A positive association was noticed between grain Zn content and grain quality traits, *viz.*, grain length, width, and weight (Zhang et al., 2004; Anandan et al., 2011). This indicates the prominence of grain quality traits in breeding for rice biofortification, in addition to the grain Zn content. The aroma was found to be closely associated with high grain Zn content, and epistatic interactions were also reported (Gregorio, 2002). The association between grain Zn content and grain yield is a key factor in identifying and releasing biofortified rice varieties. Anusha et al. (2021) found a strong positive link between grain Fe and Zn and single-plant yield in hybrids grown aerobically, but only a slight negative correlation when grown under transplanted conditions. The negative correlation between Zn content and grain yield was reported by Suman et al. (2021). Therefore, simultaneous selection for both Zn content and grain yield can be practiced for developing genotypes with high micronutrients along with high grain yield. Cantila and Quitel (2020) reported a positive and moderate correlation between Fe and Zn contents in 20 Philippine traditional rice varieties with mean Zn and Fe contents of 30.26 and 14.56 mg/kg, respectively. The high Zn traditional rice varieties should be utilized in the pre-breeding program to increase grain yield along with high Fe content. Pandey et al. (2019) reported neither significant positive nor significant negative effects of Zn and Fe contents with grain yield in different parts of rice grain. Therefore, simultaneous selection has to be made to obtain a genotype with higher grain Zn and Fe contents and higher grain yield per plant. Lee et al. (2020) reported a positive correlation between grain Zn and Fe levels, but grain Fe content and length-to-width ratio had a negative association. This suggested that enhancing both Zn and Fe contents in rice grains at the same time could improve rice grain micronutrient profile. A significant negative association between grain Zn and Fe contents and yield in rice was observed in earlier studies (Gao et al., 2006; Norton et al., 2010; Dixit et al., 2019). The reason for such a negative trend was because of the past selection toward grain yield and its favorable alleles, while no selection was performed for Zn/Fe trait in breeding programs. Therefore, fixed breeding materials generated from the crosses between high Fe/Zn and high-yield parents will describe the perfect relation between these traits. However, a positive association between grain Zn content and grain yield was observed under zinc-deficient soils with a diverse panel of aromatic rice and landraces (Gregorio, 2002). Increased seed vigor is associated with dense micronutrients in grain; higher levels of Zn and P in grains contribute to better crop establishment (Phattarakul et al., 2012; Mayamulla et al., 2017). Al-Daej (2022) revealed a significant positive association between grain Fe, Zn, and protein contents, which indicates that selection for one of the quality traits along with grain yield will result in the development of biofortified varieties with high grain yield. Calayugan et al. (2020) also reported a positive association between grain Fe and Zn contents in rice because of the common mechanism of uptake and transport. This positive genetic association between grain yield and Fe, Zn, and protein content helps the breeder in devising the right breeding strategy for the simultaneous improvement of all the desired traits. *Ghd7* controlling heading date is a negative regulator of Zn content in brown rice (Alam et al., 2020). The evidence of recent studies, including the identification of genetic resources, transgenic rice, and released varieties for high grain Zn content coupled with grain yield, provides the possible outcome in rice biofortification (TO, 2014). Positive association of the Fe and Zn with yield trait is helpful in increasing the yield simultaneously with enhanced micronutrients. The evasion of the selection of those traits, which have a negative correlation with yield, is equally important in maintaining the balance of both parameters. The negative associations can be broken down by different breeding strategies, where “biparental mating” plays a crucial role. The biofortified breeding materials and released Zn rice varieties are examples for breaking the negative linkages (selection in larger segregating generations) between Zn/Fe and grain yield traits, as they are bred for both the traits. In many of the studies, positive correlations were observed between Zn and Fe, Zn and protein, and Zn and grain yield; simultaneous selection and improvement in both the traits can be practiced to develop high-yielding biofortified rice varieties in future.

### 4.4 Biofortified rice varieties

HarvestPlus program is leading and coordinating the biofortification breeding in staple crops including rice to alleviate micronutrient deficiencies in developing countries. Biofortification adopts modern plant breeding and molecular approaches to fortify rice grains with high micronutrient content (Graham et al., 2001). Efforts have been initiated by the Department of Biotechnology-India (DBT), Golden Rice by the International Rice Research Institute (IRRI), and the Indian Council of Agricultural Research (ICAR) to support biofortification research toward the development of biofortified varieties (Sanjeeva Rao et al., 2020). In addition, HarvestPlus is a CGIAR program implemented in India in 2007 with a mission to reduce malnutrition and improve the livelihood of millions. Biofortification trial was initiated through the All India Coordinated Rice Improvement Programme (AICRIP) in 2013 to evaluate promising breeding lines with a threshold level of zinc at 20 mg/kg and revised to 24 mg/kg, which resulted in the release of a few biofortified varieties (**Table 3**) coordinated by ICAR—Indian Institute of Rice Research (IIRR) (Sanjeeva Rao et al., 2020). In addition, several biofortified enriched rice have been developed across the globe, and Bangladesh became the first country in the world to approve β-carotene-enriched golden rice (GR) variety BRRI Dhan 29 for cultivation (Glover et al., 2020).

**Table 3:** List of biofortified rice varieties released so far in the world.

S. no.	Country	Name of varieties	Parentage	Year of release	Parameter (Fe, Zn, Vit A, and protein)	Reference
1.	Philippines	NSIC Rc172 (MS 13)	IR68144-3B-2-2-3(IR72/ZawaBonday)	2003	Fe 13-21 mg/kg (brown rice)	https://agris.fao.org/
2.	Bangladesh	BRRI Dhan 62	Jirakateri/BRRI dhan39	2013	Zn 19.6 mg/kg	Swamy et al., 2016
3.	Bangladesh	BRRI Dhan 64	IR 75382-32-2-3-3/BR 7166-4-5-3-2-5-5B1-92	2014	Zn 25 mg/kg	Swamy et al., 2016
4.	Bangladesh	BRRI Dhan 72	BR 7166-4-5-3/BRRI Dhan 39	2015	Zn 25 mg/kg	Tsakirpaloglou et al., 2019; Bin Rahman and Zhang, 2023
5.	India	Chhattisgarh Zinc Rice 1	Poornima/Annada	2015	Zn 22–24 mg/kg	Yadav et al., 2020
6.	Bangladesh	BRRI Dhan 74	–	2016	Zn 24.2 mg/kg	Tsakirpaloglou et al., 2019; Bin Rahman and Zhang, 2023
7.	Bangladesh	BRRI Dhan 69	–	2016	Pro Vitamin-A and low GI 54.9	Tsakirpaloglou et al., 2019; Bin Rahman and Zhang, 2023
8.	Philippines	NSICRc460	–	2016	Zn 19.6 mg/kg	https://60.irri.org/
9.	Indonesia	INPARI IR Nutri Zinc	IR91153-AC 82/IR05F102//IR 68144-2B-2-2-3-166///IRRI145	2016	Zn 26 mg/kg	https://60.irri.org/
10.	India	DRR Dhan 45	IR 7307-45-3-2-3/IR 77080-B-34-3	2016	Zn 22.3 mg/kg	Sanjeeva Rao et al., 2020
11.	India	CR Dhan 310	Naveen/ARC 10075	2016	Protein 10.3%	Chattopadhyay et al., 2019
12.	Bangladesh	BU Aromatic Hybrid Dhan-1	KK8/Badshabhug	2016	Zn 21.8 mg/kg; Fe 10 mg/kg	http://dhcrop.bsmrau.net/bu-aromatic-hybrid-dhan-1
13.	Bangladesh	BU Aromatic Dhan-2	Basmati/IR58025B	2016	Zn 22 mg/kg; Fe 10 mg/kg	http://dhcrop.bsmrau.net/bu-dhan-2
14.	India	DRR Dhan 48	RP Bio 226*1/CSR 27	2017	Zn 20.91 mg/kg	Sanjeeva Rao et al., 2020
15.	India	DRR Dhan 49	RP Bio 226*1/CSR 27	2017	Zn 26.13 mg/kg	Sanjeeva Rao et al., 2020
16.	India	Surabhi	PRN-19045/PRN-14	2017	Zn 22.84 mg/kg	Sanjeeva Rao et al., 2020
17.	India	GR-15	Bhurarata/NAUR-1	2017	Zn 21.58 mg/kg	https://nau.in/nauvariety
18.	India	Surabhi	PRN-19045/PRN 14	2017	Zn 22.84 mg/kg	Sanjeeva Rao et al., 2020
19.	Bangladesh	Binadhan 20	Binasail/Vietnam rice	2017	Zn 26.5 mg/kg; Fe 20-31 mg/kg	http://dhcrop.bsmrau.net/binadhan-20/
20.	Bangladesh	BRRI Dhan 84	BRRIdhan29/IR68144//BRRI dhan28///BR11	2017	Zinc 27.6 mg/kg; Fe 10.1 mg/kg; protein 9.7%	Kader et al., 2020
21.	India	CR Dhan 315	Swarna/ARC 10075	2018	Zn 24.9 mg/kg	Yadav et al., 2020
22.	India	Zinco Rice-MS (R-RHZ-LI-23)	Lalmati/IR 681444B-13-2-1-1	2018	Zn 27.4 mg/kg	Yadav et al., 2020
23.	India	Chhattisgarh Zinc Rice-2	R 1243-1224-578-1	2018	Zn 22–24 mg/kg	Yadav et al., 2020
24.	India	CR Dhan 311	Naveen/ARC 10075	2019	Protein 10.1% and Zn 20.1 mg/kg	Chattopadhyay et al., 2019
25.	Indonesia	INPARI 47 Nutri Zn	IR91153-AC 82/IR05F102//IR 68144-2B-2-2-3-166///IRRI145	2019	Zn 29.54 mg/kg	https://bbpadi.litbang.pertanian.go.id/index.php/varietas-padi/inbrida-padi-sawah-inpari/inpari-ir-nutri-zinc
26.	Bolivia	CIAT BIO-44 +Zinc	–	2019	Zn 25 mg/kg	https://marlo.cgiar.org/
27.	El Salvador	CENTA A-Nutremas	–	2019	Zn 22.86 mg/kg; Fe 6.9 mg/kg	https://marlo.cgiar.org/
28.	Bangladesh	BRRI Dhan 96		2019	Protein 10.8%	Bin Rahman and Zhang, 2023
29.	Nicaragua	INTA Las Minas	–	2020	Zn 25.0 mg/kg	HarvestPlus, 2020
30.	Colombia	Fedearroz BIO Zn 035	BF14AR035	2021	Zn 26 mg/kg	https://www.en.krishakjagat.org/seed-industry/colombia-welcomes-first-biofortified-zinc-rice-variety/
31.	India	CR Dhan 411	ARC 10075/Swarna*3	2021	Protein 9.88%	https://www.icar-nrri.in
32.	India	DRR Dhan 63	IET 17280/Pusa Basmati 1	2021	Zn 24.2 mg/kg	https://www.icar-iirr.org
33.	Bangladesh	BRRI Dhan 100 (BR8631-12-3-5-P2)	BR7166-5B-5/BG305	2021	Zn 25.7 mg/kg	Kader et al., 2020; Bin Rahman and Zhang, 2023
34.	Bangladesh	BRRI Dhan 102	IR99285-1-1-1-P2	2022	Zn 25.5 mg/kg	Bin Rahman and Zhang, 2023
35.	Philippines	NSIC Rc648	–	2022	Zn 23.2 mg/kg	www.philrice.gov.ph
36.	India	DRR Dhan 67 (BRRI Dhan 84)	BRRI Dhan 29/IR 68144/BRRI Dhan 28///BR 11	2022	Zn 27.6 mg/kg	https://www.icar-iirr.org
37.	India	DRR Dhan 69 (BRRI Dhan 100)	BR 7166-5B/BG305//BRRI Dhan 29	2022	Zn 25.7 mg/kg	https://www.icar-iirr.org

To commemorate the 75th Anniversary of the Food and Agriculture Organization (FAO) on 16 October 2020, the Indian Government rolled out an ambitious *POSHAN Abhiyaan* targeting over 100 million people with the aim to reduce stunting, undernutrition, anemia, and low birth weight. The government of India dedicated 17 biofortified varieties covering eight crops and will have up to a threefold increase in nutritional value, in which rice variety CR Dhan 315 with high zinc is one of them. One of the UN’s 17 sustainable development goals is the development of nutritionally rich varieties of crops with enhanced micronutrients like iron, zinc, calcium, total protein, quality of protein with high lysine and tryptophan, anthocyanin, pro-vitamin A, and oleic acid coupled with reduced levels of anti-nutritional factors. In line with international priorities, the ICAR has produced 53 such rice varieties in the last 5 years, where only one biofortified variety was developed prior to 2014 (https://pib.gov.in/PressReleaseIframePage.aspx?PRID=1664231).

### 4.5 Anti-nutritional factors and bioavailability of micronutrients

The fraction of the total amount of vitamins or nutrients that are potentially absorbable in a metabolically active form is known as bioavailability. In plant-based foods, the absorption rate of iron and zinc is less than 10%, for example, 1% in rice (Bhatnagar and Padilla-Zakour, 2021). This is because many inhibitory factors impair iron and zinc absorption. Phytic acid (PA) is one of the major naturally occurring anti-nutrient factors that reduce the bioavailability of Fe and Zn contents and absorption in the human body. Rice is a staple meal in developing countries, but its high levels of dietary PA provide a barrier to nutritional adequacy, and the excretion of unmetabolized PA by monogastric animals causes eutrophication (Brinch-Pedersen et al., 2002). Hence, the bioavailability of Fe and Zn contents can be increased by reducing the level of PA content. Simultaneously, identifying the genotypes having high grain Zn and Fe with low PA content is essential to develop nutrition-rich rice varieties.

#### 4.5.1 Strategy to improve micronutrient availability by reducing phytic acid content

Phytic acid is an insoluble salt that strongly binds to essential micronutrients and affects their absorption in non-ruminants. The human digestive system lacks phytase, which is required to hydrolyze inositol phosphate bonds and impairs the absorption of iron, zinc, and calcium in human digestion, resulting in acute deficiency (Iwai et al., 2012). The PA content in rice bran ranges from 2.6 to 8.7 g/100 g dry weight (Lehrfeld, 1994). Improving Fe and Zn contents in brown rice will not be sufficient in alleviating hunger unless anti-nutritional factors like PA are reduced. Stangoulis et al. (2007) identified quantitative trait loci (QTLs) (qPA.12) controlling phytate content in the DH population of IR64/Azucena. An Ipa mutant showed a 45% reduction in PA content and showed an additive effect of 5.4 mg/kg zinc content availability in Kay bonnet, which is non-lethal mutant rice (Larson et al., 2000). Nagina 22-derived Pusa LPA mutant resulted in mapping a major QTL, qLPA8.1, explaining a 22.2% phenotypic variation of reduced PA (Gyani et al., 2020). It is imperative to develop cultivars with low PA content, thereby identifying potential donors and coalescence of low PA with high micronutrient content in brown rice. Thus, lowering the PA content in brown rice is one of the cost-effective sustainable approaches to increasing Fe and Zn bioavailability in rice (Gyani et al., 2020; Wang et al., 2021). Wang et al. (2021) developed double mutant lines with a combination of high Zn content and low phytic acid in grain to improve Zn bioavailability. To overcome mineral deficiency, low phytate mutants in the background of super basmati with better germination and yield advantage over the parent were also developed (Qamar et al., 2019). Tp et al. (2022) developed functional markers for low phytic acid-related candidate genes. Poor seed germination and low grain yield are reported as the breeding consequence of lowering the PA in staple crops and reducing the scope of applied breeding (Raboy et al., 2000; Pilu et al., 2005).

#### 4.5.2 Role of amino acids in enhancing the bioavailability of Fe and Zn contents

Rice protein is deficient in essential amino acids like lysine; hence, improving amino acids (sulfur-containing cysteine and particularly methionine) and promoter substances like ascorbate enhances the bioavailability of Fe and Zn contents (Hallberg, 1981; Bhullar and Gruissem, 2013). A slight increase in amino acid content may have positive effects on micronutrient bioavailability in human diet (Bouis et al., 2003). However, an increase in protein is negatively associated with grain yield, while elevating the levels of N fertilizer has positive effects on protein content in rice (Evans et al., 1980).

### 4.6 Phenotyping tools for quantification of grain Fe and Zn contents

Reliable and robust phenotyping is a prerequisite for effective estimation of micronutrient content in grains. There are several qualitative and quantitative methods that are adopted in their estimation like titrimetry, colorimetry, flame photometry, inductively coupled plasma–mass spectrometry (ICP-MS), atomic absorption spectrometry (AAS), inductively coupled plasma–atomic emission spectroscopy (ICP-OES), and energy-dispersive X-ray fluorescence (ED-XRF) spectrophotometry. Titrimetry and colorimetry are first-generation methods, which are less sensitive and require specific reaction conditions. Flame photometry is superior to these two but inferior to AAS in terms of sensitivity and interference from other substances. AAS can analyze one element at a time, while ICP can determine multiple elements and their isotopes simultaneously. Except for ED-XRF, all are destructive quantitative methods wherein seeds are subjected to three stages for iron and zinc content estimation: 1) sample processing where paddy should be thoroughly cleaned and converted to brown or polished rice; 2) preparation of sample solution under specific conditions like digestion of sample in the tri-acid or bi-acid mixture at high temperatures in sample digestion chambers; 3) analysis of sample solution in the respective equipment. In ED-XRF, only the first step is required, and thoroughly cleaned and uncontaminated brown or polished rice grains can be analyzed in the machine against a pre-loaded standardized method. The advantages of ED-XRF include the following:

a) The brown rice sample can be germinated for next-generation crops, which is a huge advantage to selecting the promising lines in the initial generations itself.
b) Similarly polished rice analyzed in this machine can be used for other analyses where the sample is destroyed.
c) In other methods, chances of contamination can increase during sample solution preparation from acids, glassware, distilled water, etc.
d) The cost of ED-XRF is cheaper than that of AAS and ICP.
e) Non-necessity of costly chemicals like highly pure iron and zinc standards, high-quality acids, and skilled human resources as well as time for digestion and analysis, making easier, with ED-XRF cheaper and more convenient than AAS or ICP.

Hence, presently, ED-XRF can be considered high-throughput equipment for iron and zinc estimation. The efficiency of ED-XRF is 200–300 samples per day. Furthermore, ED-XRF is non-destructive, is low cost, has a high resolution, and is easy to use, making it a breeder-friendly phenotyping approach (Govindaraj et al., 2016; Swamy et al., 2016; Maganti et al., 2020). Therefore, XRF benchtop spectrometers are much more cost-effective than ICP and AAS when considering the initial costs of instruments and infrastructure, as well as daily consumables. XRF is widely used in the present-day estimation of Fe and Zn contents in many crops (Govindaraj et al., 2016). In addition, care should be taken to avoid iron or zinc contamination from processing machines like dehullers and polishers, as well as iron-based material. Breeders should use ED-XRF for a large number of sample screenings and discard lower Fe/Zn materials in early generations.

## 5 Genomic approaches

In the genomics era, tremendous crop improvement efforts have benefited through advances in next-generation molecular markers, gene sequencing technology, gene editing, and other biotechnology approaches. In utilizing the vast genomic resources, there is more scope to study the nutritional improvement in crops. This allows us to identify the marker–trait associations/QTL to integrate into genomics-assisted breeding programs. In this section, we discussed the reported identification and application of QTL/genes using genomics-assisted breeding for iron and zinc biofortification in rice.

### 5.1 Biparental and backcross-derived populations

By using the molecular marker technique, several QTLs related to iron, zinc, and other nutritional quality traits have been mapped in rice (**Supplementary Table 1**). Swamy et al. (2021) mapped QTLs associated with Fe across 12 rice chromosomes; among them, 17 were identified as stable, and 25 were harboring Fe-related genes with or near the QTLs. Wild rice such as *O. nivara* and *Oryza rufipogon*, deep water rice such as Madhukar, Jalmagna, and landraces reported positive alleles responsible for Fe content in the study (Anuradha et al., 2012; Swamy et al., 2018; Swamy et al., 2021). The study by Gregorio et al. (2000) reported three loci explaining 19%–30% phenotypic variance (PV) on chromosomes 7, 8, and 9 for Fe content. Similarly, two QTLs for Fe on chromosomes 2 and 9 while three QTLs for Zn on chromosomes 5, 8, and 12 (Garcia-Oliveira et al., 2009) were also reported. Stangoulis et al. (2007) identified two QTLs for Zn on chromosomes 1 and 12; three QTLs for Fe on chromosomes 2, 8, and 12; and a common QTL for Fe and Zn.

**Figure 4:**
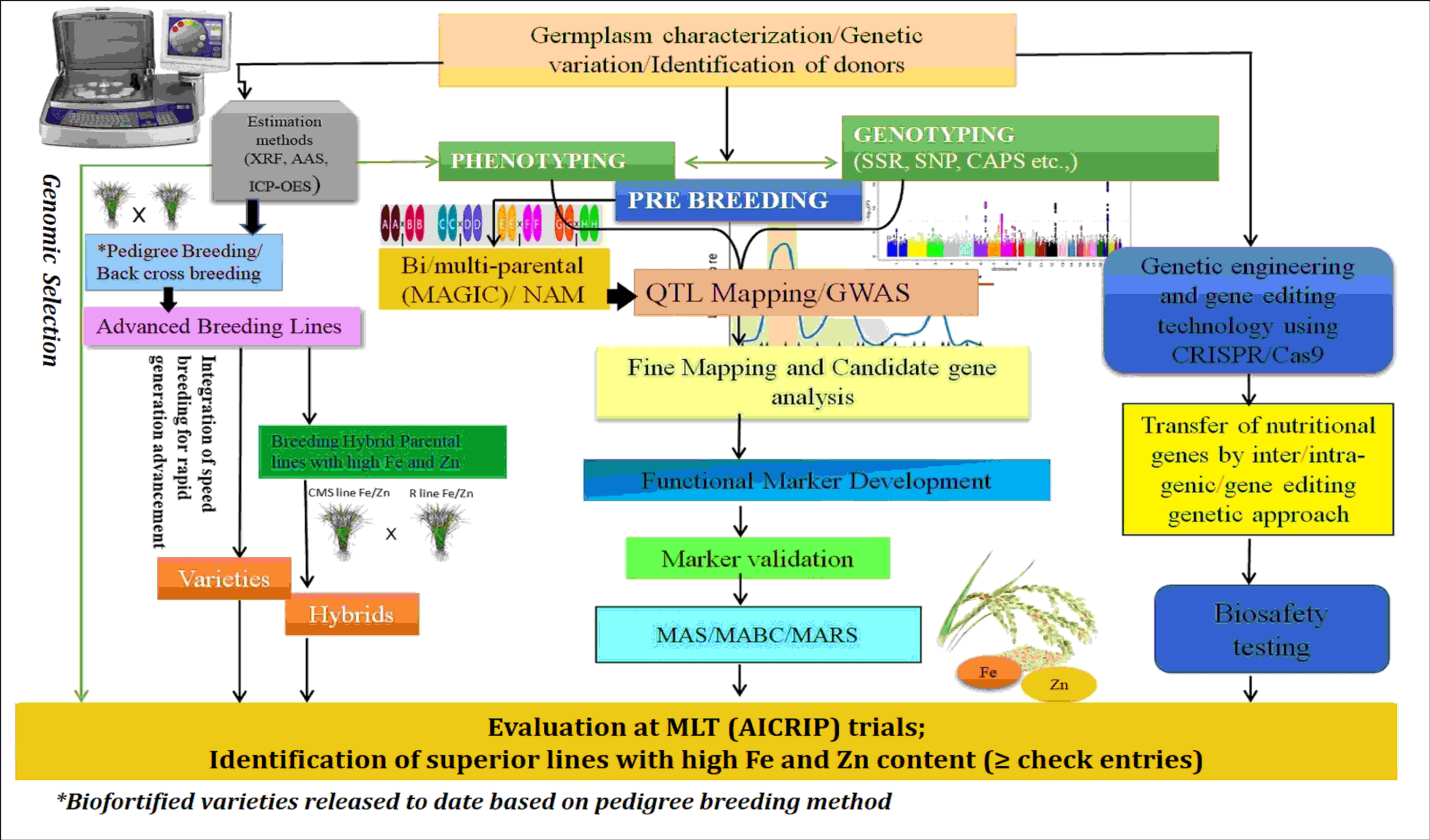
Integrated breeding approaches for rice biofortification. AAS, atomic absorption spectroscopy; XRF/ED-XRF, energy-dispersive X-ray fluorescence; ICP-OES, inductively coupled plasma–optical emission spectroscopy; QTL, quantitative trait loci; GWAS, genome-wide association study; AICRIP, All India Coordinated Rice Improvement Programme; MLT, Multi Locational Trial; CRISPR/Cas9, Clustered Regularly Interspaced Short Palindromic Repeats, CRISPR-associated proteins9 technology; SSR, simple sequence repeats; SNP, single-nucleotide polymorphism; CAPS, cleaved amplified polymorphic sequences; MAS, marker-assisted selection; MABC, marker-assisted backcrossing; MARS, marker-assisted recurrent selection.

The population derived from the parents high-yielding PAU 201 and iron-rich variety Palman 579 showed transgressive segregation for grain Fe and Zn, with higher Fe (475 μg/g) and zinc (157 μg/g) percentages. The mapping study on this population reported 11 putative QTLs for Fe and three QTLs for Zn in rice grains located on chromosomes 2, 3, 7, 10, and 12 (Kumar et al., 2014). Dixit et al. (2019) derived a population from parents RP-Bio 226 and Sampada to locate genomic regions associated with grain Fe and Zn contents and found that they shared the same genomic regions on chromosome 1. Swamy et al. (2018) developed BC_2_F_3_ mapping populations derived from the crosses of Swarna with *O. nivara* accessions to map QTLs for Fe and Zn contents. From this study, QTLs via *qFe2.1*, *qFe3.1*, *qFe8.2*, and *qZn12.1*, were consistently found in both populations and represented more than 15% of phenotypic variance. Islam et al. (2020) investigated 113 aromatic rice germplasm and mapped QTL (*QTL.Fe.9*) for Fe content on chromosome 9, and two (*QTL.Zn.4* and *QTL.Zn.5*) QTLs for Zn on chromosomes 4 and 5 were detected. Mohiuddin et al. (2020) mapped QTLs associated with higher levels of Zn in rice grain in an F_2_ population derived from a cross between a high-yielding variety, BRRI Dhan 28, and a locally adapted Zn-enriched cultivar, Kalobokri; and they detected a large-effect QTL, *qGZn3*, on chromosome 3 between RM5419 and RM1164 markers. In the interval between ah03002520 and cmb0336.5 on chromosome 3, two co-localized QTLs, *qFe3-1* and *qZn3-1*, were discovered in a double-haploid population developed from the rice cultivars (93-11 and Milyang 352) for iron and zinc (Lee et al., 2020). Suman et al. (2021) mapped 13 QTLs from 190 recombinant inbred line (RIL) populations derived from PR116 and Ranbir Basmati by using 102 simple sequence repeats (SSRs) and 21 QTLs by using genotyping by sequencing (GBS) data of 44 RILs. Among these, two major QTLs (*qZPR.1.1*-PV, 37.84%; *qZPR.11.1*-PV, 15.47%) for grain Zn in polished rice and a common QTL (*qZBR.2.1* and *qZPR.2.1*) for grain Zn content in brown and polished rice were identified through SSRs and single-nucleotide polymorphism (SNP) genotyping. Pippal et al. (2021) identified QTLs for high Fe and Zn contents from the RILs of F_5_ and F_6_ generations derived from PAU 201 and Palman 579 and detected 16 QTLs altogether for grain Fe, Zn, and single-plant yield traits. The generic comparison of unique breeding and molecular approaches for enhancing micronutrient availability in final products is depicted in **Figure 4**. However, this integrated approach should be customized based on the additive on non-additive genetics of the micronutrient traits and by adopting appropriate breeding strategies (heterosis breeding and pure line/recurrent selection). In rice, several studies reported the predominance of additive genetic variances for Zn/Fe, indicating that progeny-test-based selection is likely to be more effective. This type of selection will help the breeders to improve the elite parental population toward higher Zn/Fe content in addition to its agronomic traits. Subsequently, planned crosses involving high × high (for Zn/Fe) may reward progenies having better micronutrient content than the parent that contains the least amount of micronutrient (if additive operates), but it is wise to select progenies that exceeds mid-parent values. In contrast, the micronutrient content in the progenies may exceed than that in their parents that contain high micronutrient content (if non-additive operates). In both cases, considering that established nutrient baseline levels and breeding targets are very critical, selection of progenies above the baseline checks is highly recommended. The use of robust phenotyping methods aids in the discovery of possible KASP markers (SNPs), such as *NAS3*, which is essential to enhance marker-based selection of micronutrient traits in rice.

### 5.2 MAGIC population

Descalsota et al. (2018) conducted a genome-wide association study (GWAS) using 144 multi-parent advanced generation inter-cross (MAGIC) plus lines to identify QTLs and SNP markers for biofortification. *OsMTP6*, *OsNAS3*, *OsMT2D*, *OsVIT1*, and *OsNRAMP*7 control iron and zinc homeostasis and were found to be co-located with QTLs. Several QTLs/gene pyramided lines having both high zinc and yield potential that serve as good sources in breeding programs were also identified, and they can also be released as biofortified varieties after evaluation. Liu et al. (2021) conducted GWAS using 212 MAGIC populations developed by four parents and identified five QTLs associated with Zn content in the shoot and six QTLs associated with Zn content in brown rice at the mature stage.

### 5.3 Genome-wide association studies

GWAS is a powerful tool to identify the genomic regions associated with traits of interest utilizing the high-density marker system. Bollinedi et al. (2020) studied 192 Indian rice germplasm accessions, which resulted in the identification of 29 significant marker–trait associations (MTAs) for traits such as FeBR (6 MTAs), FeMR (7 MTAs), ZnBR (11 MTAs), and ZnMR (5 MTAs) using FarmCPU model showing the phenotypic variation ranging from 2.1% to 53.3%. Cu et al. (2021) reported 12 elements including Fe and Zn, identified 128 loci for grain elements and 57 loci for agronomic traits, and revealed potential candidate genes including *OsNAS3*, which control Zn content by screening 233 indica germplasm. In addition, they identified consistent QTLs across environments, especially for Zn (*qZn7.2*), reported its stability in different genetic backgrounds, and suggested its usage in breeding programs for Zn biofortification. Descalsota et al. (2018) reported two QTLs for iron and five QTLs for zinc in 152 colored rice diversity panels. Three of the seven QTLs were co-located with the metal homeostasis genes, *viz.*, zinc ion transporter, *OsCNGC16*, *OsDof*, *OsHMA9*, *OsNRAMP3*, *OsbZip85*, *OsNRAMP7*, and *ZFP252*. In another study, novel QTLs for grain Fe (*qFe3.3* and *qFe7.3*, *qZn2.2*, *qZn8.*3, and qZn12.3), and Zn content were detected; in addition, four QTLs (*qFe3.3*, *qFe7.3*, *qFe8.1*, and *qFe12.2*) for grain Fe content were found to be co-localized with QTLs controlling grain zinc content (*qZn3.1*, *qZn7*, *qZn8.3*, and qZn12.3). Some Fe–Zn-controlling QTLs were co-localized with the yield component QTLs *qTBGW*, *OsSPL14*, and *qPN*. Therefore, simultaneous improvement of Fe and Zn along with yield component traits is possible (Pradhan et al., 2020).

### 5.4 Transgenic approaches

The transgenic approach acts as a sustainable and efficient tool for enhancing the nutritional quality traits when the required trait of interest is not available in existing germplasm (Lucca et al., 2001; Zimmermann and Hurrell, 2002). Multiple reports show that expression of certain genes like nicotinamine aminotransferase, iron transporter *OsIRT1*, nicotinamine synthase 1 (*OsNAS1*) and 2 (*OsNAS*2), soybean ferritin, and common bean ferritin can increase iron content. Similarly, overexpressing *OsIRT1* (49) and mugineic acid synthesis genes from barley [*HvNAS1*, *HvNAS1*, *HvNAAT-A*, *HvNAAT-B*, and *IDS3*] will enhance zinc content to different levels. Expression of ferritin genes *nicotinamine synthase genes* (*NAS*) or *ferritin* in conjunction with *NAS genes* will increase the Fe content of rice endosperm ranging from twofold increase via single-gene approaches to sixfold increase via multi-gene approaches. Synergistic effect of *nicotinamine synthase*, *ferritin*, *and phytase genes* (*NFP*) in transgenic rice noted a sixfold increase in iron content in polished rice (Wang et al., 2013). Nicotinamine synthase (*OsNAS2* gene) is an effective promoter in iron utilization as well as its fortification in polished rice and thus has a huge potential in combating iron deficiency (Zheng et al., 2010).

By expressing the soybean ferritin gene, *SoyferH1*, in endosperm using the rice endosperm-specific 1.3-kb *OsGluB1* promoter, Goto et al. (1999) generated transgenic rice plants that showed threefold (38.1 ± 4.5 µg/g DW) higher levels of Fe accumulation in the endosperm. Similarly, Lucca et al. (2001) increased the iron content by two times (11.53 ± 0.16 and 22.07 ± 0.70 µg/g per seed) in rice endosperm by introducing a ferritin gene from *P. vulgaris* into rice grains. They have introduced a thermo-tolerant phytase from *Aspergillus fumigatus* into the rice endosperm to increase iron bioavailability and indicated that this rice has the potential to significantly boost iron nutrition in populations, where iron deficiency is widespread, but the phytase did not withstand cooking. Vasconcelos et al. (2003) developed a high-iron (IR68144) rice variety by overexpressing the soybean ferritin gene, which increased iron content by 3.7-fold in polished rice grains. Transgenic plants with enhanced levels of tolerance to low-Fe availability for calcareous soils were developed (Takahashi, 2003) with the expression of *barley nicotinamine aminotransferase* (*NAAT*) *gene*. The overexpression of *OsSUT1* promoter-driven *OsYSL2* gene was found effective for iron biofortification. Its overexpression of *OsYSL2* gene increases the iron content by fourfold under the *sucrose transporter* (*OsSUT1*) promoter, and its disruption decreases iron content by 18% in brown rice and by 39% in polished rice in comparison with the control plants. The importance of the *OsYSL2* gene was demonstrated in rice (Ishimaru et al., 2007). Similarly, overexpression of *OsNAS1*, *OsNAS2*, and *OsNAS3* increased the content of nicotinamine, Fe, and Zn in three populations of rice generated by Johnson et al. (2011). Selected lines from each population had up to a fourfold increase in Fe content in polished grains. Twofold increases in Zn content were also observed in the *OsNAS2* population in comparison with the control plants.

Iron storage protein ferritin increases the iron storage capacity in the grains under the control of endosperm-specific promoters. Using this approach, Theil et al. (2012) achieved a twofold increase in the Fe content in polished grains of transformants. Iron stored in ferritin is an important source for humans to avoid iron deficiency. *Ferritin gene* (*Osfer2*) was overexpressed in PSII rice, which accumulated 2.09-fold of Fe and 1.37-fold of Zn (Paul et al., 2012). RNAi-mediated silencing of *MIPS* gene of the phytic acid metabolism pathway increased the content of iron, zinc, calcium, and magnesium in milled rice grain (Ali et al., 2013). An increase in iron content of 10.46 μg/g dry weights in polished rice grain was achieved by the combined expression of four genes *AtIRT1* with *Pvferritin*, *AtIRT21*, and *AtNAS1* genes (Boonyaves et al., 2016). Simultaneous enhancement of iron and zinc along with β-carotene was performed in endosperm of rice lines by expressing *Arabidopsis Nicotianamine synthase 1* (*AtNAS1*), *bean Ferritin* (*PvFERRITIN*), *bacterial Carotene desaturase* (*CRTI*), and *maize Phytoene synthase* (*ZmPSY*) in a single genetic locus (Singh et al., 2017). Fe homeostasis genes *OsNAS1*, *OsNAS2*, *OsFer*, *OsVIT1*, *OsVIT2*, *OsZIP*, *OsIRO2*, and *OsIRT1* were already cloned, and transgenic plants have been developed, which had shown sixfold and fourfold increase in Fe and Zn contents, respectively (Kawakami and Bhullar, 2018; Swamy et al., 2021).

### 5.5 Candidate gene analyses

The candidate genes with predicted function serve as an important source to generate novel molecular markers within a given QTL region. These genes have more possibility to show stable association across the mapping populations or genetic stocks and will provide a useful tool for the isolation and molecular characterization of QTLs (Kearsey, 1998). The cause-and-effect relationship between the particular QTL and a nearby candidate gene mapping was used in this approach (Tuberosa and Salvi, 2007). Putative CGs belonging to five gene families, *viz.*, *OsYSLs*, *OsNRAMPs*, *OsZIPs*, *OsFROs*, and Ferritin, have been identified as metal transporters in rice (Gross et al., 2003). Along with these, NAS genes and *OsHMA7* are potential candidate genes for Fe and Zn biofortification in rice (Swamy et al., 2021). Two QTLs for iron and five QTLs for zinc were identified (Chandel et al., 2011). They have also identified eight CGs that are predicted to be involved in activities of uptake, transport, and accumulation of Fe and Zn in rice. Four candidate genes (*OsARD2*, *OsIRT1*, *OsNAS1*, and *OsNAS2*) *for Zn*, two (*OsYSL1* and *OsMTP1*) for Fe, two (*OsNAS3* and *OsNRAMP1*) for heavy metal ion transport, and one (APRT) for both Fe and Zn were identified from 12 QTLs while studying 168 RILs derived from Madhukar × Swarna (Anuradha et al., 2012). Variation in nucleotide sequence at primer binding sites within the six putative candidate genes (*OsNAC5*, *OsZIP1*, *OsNAC*, *OsYSL2*, *APRT1*, and *OsNRAMP1*) was the probable causal factor for differential grain zinc and iron accumulation in rice Kumari et al. (2018). Swamy et al. (2018) discovered 10 and eight QTLs in one BC_2_F_3_ population as well as seven and five QTLs in another BC_2_F_3_ population 2 for grain Fe and Zn contents, respectively. Jeong et al. (2020) discovered 21 QTLs related to Fe and Zn. The major-effect QTLs *qFe7* and *qZn7* provided the highest contribution to phenotypic variance for grain iron and zinc contents. Eleven candidate genes related to Fe- and Zn-related candidate genes were found in close proximity to *qFe7*, *qZn7*, and chromosome 7, with an additive effect on iron content. Sixteen candidate genes for metal homeostasis were discovered to be co-located with 10 QTLs for Fe and Zn contents in a double-haploid population produced from an intra-*japonica* hybrid between ‘Hwaseonchal’ and ‘Goami 2’ (Jeong et al., 2020).

Calayugan et al. (2020) investigated a double-haploid population produced from IR05F102/IR69428 and detected two Fe QTLs (*qFe9.1 and qFe12.1*) and four zinc QTLs (*qZn5.1*, *qZn9.1*, *qZn12.1*, and *qZn12.1*). On chromosomes 9 and 12, candidate genes *OsLysM-RLK10* and *OsSWEET13* were discovered as QTLs for Fe, whereas candidate genes *OsGATA8*, *OsSar1b*, *OsZIP6*, and *Os09g0511500* on chromosomes 1, 5, and 9 were identified as QTLs for Zn. From PR116 and Ranbir Basmati, Suman et al. (2021) generated and evaluated 190 RILs. For grain Zn in polished rice, a major QTL *qZPR.1.1* (PV 37.84%) and another QTL *qZPR.11.1* (PV 15.47%) were discovered. SSR and SNP maps revealed a common major QTL (*qZBR.2.1* and *qZPR.2.1*) on chromosome 2 for grain Zn content. Based on network analysis in the genomic regions of QTL 3 Mb, two putative candidate genes related to transporters were identified.

The grain zinc content and candidate gene markers were evaluated by Naveen et al. (2014), who reported the RILs derived from IRRI38 × Jeerige Sanna and validated the putative candidate gene markers with rice accessions. Grain zinc content was in the range of 16.1 to 35.5 mg/kg with an average of 23.7 mg/kg. Four candidate gene markers (*OsNAC*, *OsZIP8a*, *OsZIP8c*, and *OsZIP4b*) showed substantial variation among RILs, with phenotypic variations of 4.5%, 19%, 5.1%, and 10.2%, respectively. Nawaz et al. (2015) reported many Fe-binding genes and transporters, *viz.*, *OsZIP3* on chromosome 4; *OsZIP5* and *OsZIP9* on chromosome 5; and *OsNAS3* and *Peroxidase* on chromosome 6.

### 5.6 CRISPR/Cas9 system

Clustered Regularly Interspaced Short Palindromic Repeats (CRISPR) is a unique genome editing tool gaining popularity for effective targeted gene editing. In the rice genome, up to 46 target sites were edited, with an average of 85.4% mutation frequency (Ma et al., 2015). The potential examples to use CRISPR/Cas9 system are to knock out five rice carotenoid catabolic genes (*OsCYP97A4*, *OsDSM2*, *OsCCD4a*, *OsCCD4b*, and *OsCCD7*) to increase β-carotene accumulation in rice endosperm (Yang et al., 2017) and to knock down *OsVIT2* to achieve the increase of grain Fe. For increased Fe availability, the *OsVIT2* gene was knocked down through genome editing (Ludwig and Slamet-Loedin, 2019). Knocking out of *OsITPK1-6* gene through CRISPR-CAS9 leads to low phytic acid accumulation in rice grain and a consequent increase in micronutrient availability (Jiang et al., 2019).

## 6 Barriers and avenues in micronutrient breeding

### 6.1 Barriers in micronutrient breeding

#### 6.1.1 Gene × environment

Grain zinc and yield are polygenic inherited traits and highly influenced by environments (Anuradha et al., 2012). Agronomic management and genotype environmental interactions play a crucial role in identifying superior stable lines for Fe and Zn contents and have an influence on mineral uptake, translocation, and distribution to different plant parts (Phuke et al., 2017). Identifying donors from multi-location data with consistent positive correlations between Fe and Zinc with grain yield shall allow breeders to apply suitable breeding methods and strategies for micronutrient improvement (Naik et al., 2020).

#### 6.1.2 Policy regulations

In external grain fortification and agronomical management, breeding for micronutrients has its own limitation, and the use of recombinant transgenic and gene editing technology has added advantage with breeding crops in bringing micronutrient bioavailability. Few countries are successful in the release of Fe- and Zn-enriched rice varieties; such initiatives could be taken to promote and commercialization of biofortified GM varieties. It should pass regulatory standards, streamlining proper approval of biofortified GM crops, as food and feed could benefit the undernourished large sector of the community and is also a plausible antidote for hidden hunger problem.

### 6.2 Avenues in micronutrient breeding

#### 6.2.1 Employing advanced breeding approaches

The majority of biofortified rice varieties are developed using traditional breeding methods; however, success is limited due to the involvement of many QTLs with small effects, which interact highly with environmental factors (Pradhan et al., 2020). Mapping QTLs using biparental mapping populations resulted in few successes in detecting genes controlling Fe and Zn contents. Precision breeding with marker-assisted backcross or MAGIC approach by identification and introgression of genomic regions for Fe and Zn is a better approach for the enhancement of desired micronutrients in popular rice varieties. Parallel breeding of a large set of germplasm through association mapping has positive output in terms of high resolution, less cost, and time-saving when compared with conventional breeding techniques (Pradhan et al., 2020). Nested association mapping (NAM) is one of the effective strategies to identify novel genomic regions controlling micronutrient content from unexplored landraces or exotic germplasm. The use of system biology tools and approaches to analyze large amounts of genomics data is highly required in developing models for biological studies (Sauer et al., 2007). Assessment of micronutrients at early generations is not possible because of segregation for the trait, and pedigree breeding takes 4–5 years to have a stabilized population. With the advent of rapid generation advance (RGA), the so-called speed breeding will help to reduce the breeding cycle time by up to six seasons per year and will gear up for improving a large population with micronutrient traits. Currently, the established facility at International Rice Research Institute, Philippines, will have four to five generations in a year against only one to two generations under normal conditions. Estimates have shown that speed breeding can reduce the development of new varieties by 2 to 4 years.

#### 6.2.2 *De novo* domestication accelerates breeding of micronutrient-rich rice varieties

Modern gene editing technologies make it feasible to selectively domesticate genes from wild species to attain agronomically useful traits and nutritional quality at the same time (Gasparini et al., 2021). Backtracking useful genes by pan-genome sequencing that have contributed to present-day modern varieties, further editing those orthologs in wild rice and screening of segregating populations for new ideotypes are feasible *de novo* domestication (dnD) approach (Gasparini et al., 2021). Through the ages, the domestication of major food crops like rice resulted in macronutrient-rich but poor micronutrient-poor crops (Fernie and Yan, 2019). The domestication syndrome (commonly occurred traits upon domestication of diverse species) in rice resulted in the acquisition of *amino acid transporter* (*Bh4*), *Gibberellic biosynthetic enzyme* (*SD1*), *seed color* (*Rc*), *zinc finger protein* (*Sdr4*), and *seed shattering* (*qSH1*) (Kumar et al., 2021).

## 7 Future perspective

Rice biofortification research and development has shown that Fe is primarily associated with phytic acid, while Zn is bound to proteins, implying that Fe and Zn in cereal grain tissues have different evolutionary lineages. For efficient Fe and Zn biofortification strategies in cereals, future research is required to tap the resources developed in conventional breeding to elucidate the advanced molecular aspects of bivalent metal speciation, including Fe and Zn, in different tissues of seeds. Primers may be designed on the basis of loci detected, and these primers can be further utilized for the selection and improvement of the rice for Zn content in the endosperm. The two identified markers (*OsZIP7-2 and OsZIP2-1*) can be used for initial diagnostic purposes in segregating breeding populations; however, they need to be explored in-depth, and possible transgenic can be made using these genes by utilizing endosperm-specific promoters to enrich grains with Zn.

Increased comprehensive knowledge of rice genome sequencing data should lead to more useful biofortified rice in the future, as technical advancements in plant genetic modification have become a dynamic process. A further move in developing biofortified germplasm may be fine mapping of candidate genes linked to various QTLs. As SNPs were identified through next-generation sequencing (NGS), the rapid shift of research toward the utilization of CRISPR/Cas9 systems for targeted mutagenesis could be a promising approach for overcoming barriers to breeding improved-quality rice. The application of genomic selection coupled with speed breeding tools should enhance the development of biofortified varieties. Selection for high grain yield combined with the traits of high grain iron and zinc contents with the potential of plant breeding techniques could be aided by candidate gene markers. The identified genomic/QTL regions can be mined to identify the genes governing the micronutrient content, and the development of marker systems would facilitate the selection, introgression, and improvement of micronutrient content in rice. Identification of global donors for high Fe and Zn contents and development of multiparent population should be evaluated in multi-location trials to know Genotype × Environment × Management (GEM) and release of biofortified rice varieties. CGIAR rice breeding program implemented the product profile-based breeding pipeline development, which included the Fe and Zn as traits of interest for specific market segments in Asia, Africa, and Latin America and the Caribbean (LAC) (https://www.cgiar.org/research/program-platform/rice/). Despite the array of biofortified varieties that have been made available, it is urgently necessary to increase consumer access to newly released biofortified rice varieties through improved seed chain accessibility.

Breeding for nutrition should consider the post-harvest nutrition losses in order to ensure that the added nutrients bred into biofortified rice are retained during storage, processing, and cooking. For instance, under normal processing conditions, zinc rice can provide over 50% of the daily zinc needs for children. Some zinc is lost during the normal processing (milling) of rice; however, traditional, small-scale (community-based) milling tends to retain most of the germ of rice. Heavily milled, highly polished (very white) rice loses much zinc. In countries where parboiled rice is preferred (like Bangladesh), the zinc content of rice can be significantly reduced (up to 65%) because parboiling moves zinc to the outer part of the grain, which is removed by milling when producing white rice. To mitigate this, parboiled rice should preferably only be slightly milled and eaten as brown rice. If white rice is preferred, aim for only 8% to 12% bran removal (“adequate milling”) to retain a positive impact on zinc intake. The way people eat rice is heavily influenced by culture and can be hard to change, and that would be a limitation for scaling up.

## 8 Conclusions

Considering the population growth of 25% by 2050, rice production and its consumption demand continued increase in traditional areas to address food insecurity. Providing energy without adequate nutrition to people may not fulfill the food security task in the 21^st^ century. Thus, adding target nutrition in existing and newly developed rice cultivars will be a sustainable way to address food insecurity and micronutrient malnutrition and is contributing to achieving SDG goals. As long as national and international rice programs include nutrition qualities in the crop product profile as key breeding components, established nutrition trait genetics and their genetic improvement using modern phenotyping and genomic techniques undoubtedly accelerate genetic gain for these traits. The recent development in AICRP-Rice for revising the Zn standards in national testing is 24 mg/kg of polished rice (86% of Zn rice target); moreover, other agronomic traits will motivate rice breeding centers to come forward to invest in nutrition breeding. The genetics of these nutrient traits is controlled by oligo genes with higher heritability and negligible influence of G × E, implying greater selection efficiency of selection compared to complex traits like drought or yield. Thus, global biofortification efforts yielded 29 varieties rich in Zn content (16 endorsed from HarvestPlus), four rich in protein, and one each rich in high Fe and pro-vitamin A across the globe to date and also key source materials for Zn and Fe. The next generation of rice biofortification via transgenic RNAi-mediated silencing of anti-nutrient pathways and CRISPR/Cas9 system led to the development of rice varieties that showed a 6.3-fold increase in Fe and a twofold increase in Zn in rice grains. Compatible QTLs/genes play a key role in developing a desirable genotype with superior grain quality traits. By combining high-throughput genomic and transgenic-aided breeding technologies with conventional breeding, the comprehensive review will be useful in creating rice cultivars that are nutrient-dense. Rice that is bred to be high in zinc (28 mg/kg) can provide up to 60% of daily zinc needs and contribute to a reduction in zinc deficiency in regions where daily rice consumption is high such as in Bangladesh (698 g), Indonesia (479 g), Philippines (450g) and India (~ 200 g). HarvestPlus works in partnership with the International Food Policy Research Institute (IFPRI), Washington, and the International Center for Tropical Agriculture (CIAT), Columbia, to support National Agricultural Research partners and interested seed companies to breed, test, and release varieties of zinc rice in countries where rice is a key staple food. The future breeding focus must be on introducing nutrient-dense, climate-smart staples, in addition to targeting higher yields. Biofortification could be one of the solutions to climate-proof food systems addressing food and nutritional insecurity.

## Author contributions

SP, PG, and MG conceived, planned, and wrote the manuscript. JC, JV, BP, SL, SK, SD, AJ, and HA corrected and improved the genetics and breeding for biofortification aspects. NC and SR improved the biotechnological role in biofortification and conducted the experiments. SD, SS, and GR corrected the biochemical aspects of nutrition and physiological aspects. MG and PG coordinated and edited the final draft of the manuscript. All authors approved the manuscript.
