# Supplementary material for: Rice biofortification: breeding and genomic approaches for genetic enhancement of grain zinc and iron contents
Source: Front Plant Sci. 2023 Jun 2;14:1138408. doi: 10.3389/fpls.2023.1138408 (PMC10272457; doi:10.3389/fpls.2023.1138408)
Supplement: Supplementary file 1 [file Table_1.docx]

**Supplementary Table 1: List of different QTLs reported for Fe and Zn content in different mapping population of rice**

| **S. No.** | **Grain traits** | **Chr** | **QTLs** | **Markers** | **PVE range (additive effect QTLs)** | **Population Type** | **Mapping population** | **Reference** |
| --- | --- | --- | --- | --- | --- | --- | --- | --- |
|  | Fe | 1 | *qFe1.1* | RM243-RM488 | 69.0 | RILs(168) | Madhukar/Swarna | Anuradha et al., 2012 |
|  | Fe | 1 | *qFe1.2* | RM488-RM490 | 69.2 | RILs(168) | Madhukar/Swarna | Anuradha et al., 2012 |
|  | Fe | 1 | *qFe.1* | RM259-RM243 | 12.9 | RILs (241) | Zhenshan 97/Minghui 63 | Kaiyang et al., 2008 |
|  | Fe | 2 | *qFe2-1* | RM6641 | 7.0 | ILs (85) | *O. rufipogon*/Teqing | Garcia-Oliveira et al., 2008 |
|  | Fe | 2 | *qFe.2* | RM53-RM300 | 16.5 | DHs(129) | IR64/Azucena | James et al., 2007 |
|  | Fe | 2 | *qFe.2* | RM452 |  | LT/TL-RILs | Lemont/TeQing | Zhang et al., 2014 |
|  | Fe | 7 | *qFe7.1* | RM234-RM248 | 35.0 | RILs (168) | Madhukar/Swarna | Anuradha et al., 2012 |
|  | Fe | 8 | *8 qFe.8* | RM137-RM325A | 18.3 | DHs(129) | IR64/Azucena | Kaiyanget al., 2008 |
|  | Fe | 11 | *qFe.11* | RZ536-TEL3 |  | RILs (241) | Zhenshan 97/Minghui 63 | Kaiyanget al., 2008 |
|  | Fe | 12 | *qFe.12* | RM270-RM17 | 13.8 | DHs(129) | IR64/Azucena | Stangoulis et al., 2007 |
|  | Fe | 12 | *qFe12.1* | RM17- RM260 | 71 | RILs(168) | IR05F102 / IR69428 | Calayugan et al 2020 |
|  | Fe | 12 | *qFe12.2* | RM260-RM7102 | 34.0 | RILs(168) | Madhukar/ Swarna | Anuradha et al., 2012 |
|  | Fe | 5 | *qFe5.1* | RM574-RM122 | 69.2 | RILs(168) | Madhukar/ Swarna | Anuradha et al., 2012 |
|  | Zn | 1 | *qZn.1* | RM34-RM237 | 34.0 | DHs (129) | IR64/Azucena | James et al., 2007 |
|  | Zn | 5 |  | RM421 |  | LT/TL-RILs | Lemont/TeQing | Zhang et al., 2014 |
|  | Zn |  | *qZn.6* | RZ398-RM204 | 5.08 | RILs (241) | Zhenshan 97/Minghui 63 | Kaiyanget al., 2008 |
|  | Zn | 7 | *qZn7.3* | RM501-OsZip2 | 29.0 | RILs (168) | Madhukar/ Swarna | Anuradha et al., 2012 |
|  | Zn | 8 | *8qZn8-1* | RM152 | 18.0 | ILs(200) | *O. rufipogon/*Teqing | Garcia-Oliveira et al.,2008 |
|  | Zn | 8 | *qZn.8* | RM25-R1629 |  | RILs (241) | Zhenshan 97/Minghui 63 | Kaiyanget al., 2008 |
|  | Zn | 12 | *qZn.12* | RM235-RM17 | 12.8 | DHs(129) | IR64/Azucena | Stangoulis et al., 2007 |
|  | Zn | 12 | *qZn12.2* | RM260-RM7102 | 34.0 | RILs(168) | Madhukar/ Swarna | Anuradha et al., 2012 |
|  | Fe | 2 | *qFE2.1* | RM53–RM521 | 21.4 | F_2_ (247) | PAU20/Palman 579 | Kumar et al., 2014 |
|  | Fe | 2 | *qFE2.2* | RM263–RM221 | 6.9 | F_2_(247) | PAU201/Palman 579 | Kumar et al., 2014 |
|  | Fe | 2 | *qFE2.3* | RM221–RM208 | 26.8 | F_2_(247) | PAU201/Palman 579 | Kumar et al., 2014 |
|  | Fe | 3 | *qFE3.1* | RM489–RM7 | 8.8 | F_2_(247) | PAU201/Palman 579 | Kumar et al., 2014 |
|  | Fe | 7 | *qFE7.1* | RM481–RM418 | 2.4 | F_2_(247) | PAU201/Palman 579 | Kumar et al., 2014 |
|  | Fe | 10 | *qFE10.1* | RM474–RM184 | 9.2 | F_2_(247) | PAU201 /Palman 579 | Kumar et al., 2014 |
|  | Fe | 10 | *qFE10.2* | RM228–RM496 | 18.1 | F_2_(247) | PAU201 /Palman 579 | Kumar et al., 2014 |
|  | Fe | 12 | *qFE12.1* | RM491–RM519 | 16.9 | F_2_(247) | PAU201/Palman 579 | Kumar et al., 2014 |
|  | Zn | 2 | *qZN2.1* | RM521–RM29 | 5.1 | F_2_(247) | PAU201 /Palman 579 | Kumar et al., 2014 |
|  | Zn | 10 | *qZN10.1* | RM474–RM184 | 19.1 | F_2_(247) | PAU201 /Palman 579 | Kumar et al., 2014 |
|  | Zn | 10 | *qZN10.2* | RM496–RM591 | 4.7 | F_2_(247) | PAU201/Palman 579 | Kumar et al., 2014 |
|  | Fe | 01 | *qFe1.1* | RM562-RM11943 | 17.1 | BC_2_F_5_ (111) | RP Bio226/ Sampada | Dixit et al., 2019 |
|  | Fe | 01 | *qFe1.2* | RM294A-RM12276 | 14.0 | BC_2_F_5_(111) | RP Bio226/ Sampada | Dixit et al., 2019 |
|  |  | 06 | *qFe6.1* | RM8226-RM400 | 6.6 | BC_2_F_5_(111) | RP Bio226/ Sampada | Dixit et al., 2019 |
|  |  | 06 | *qFe6.2* | RM400-RM162 | 5.1 | BC_2_F_5_(111) | RP Bio226/ Sampada | Dixit et al., 2019 |
|  | Zn | 01 | *qZn1.1* | RM294A-RM12276 | 14.3 | BC_2_F_5_(111) | RP Bio226/ Sampada | Dixit et al., 2019 |
|  | Zn | 06 | *qZn6.1* | RM8226-RM400 | 34.2 | BC_2_F_5_(111) | RP Bio226/ Sampada | Dixit et al., 2019 |
|  | Zn | 06 | *qZn6.2* | RM400-RM162 | 2.9 | BC_2_F_5_(111) | RP Bio226/ Sampada | Dixit et al., 2019 |
|  | Fe | 3 | *qFe3.3* | RM7 | 8.8 | A population Panel (102 genotypes) | Biofortified lines and checks | Pradhan et al., 2020 |
|  | Fe | 7 | *qFe7.3* | RM1132 | 9.03 | A population Panel (102genotypes) | Biofortified lines and checks | Pradhan et al., 2020 |
|  | Zn | 2 | *qZn2.2* | RM300 | 11.9 | A population Panel (102 genotypes) | Biofortified lines and checks | Pradhan et al., 2020 |
|  | Zn | 8 | *qZn8.3* | RM80 | 11.86 | A population Panel (102 genotypes) | Biofortified lines and checks | Pradhan et al., 2020 |
|  | Zn | 12 | *qZn12.3* | RM260 | 5.9 | A population  Panel(102genotypes) | Biofortified lines and checks | Pradhan et al., 2020 |
|  | Fe | 6 | *qFe6* | RM3– RM340 | 18.3 | BIL(401) | IR75862(DP)/ Ce258 and ZGX1(RP) | Xu et al., 2015 |
|  | Fe | 7 | *qFe7* | RM134– RM1132 | 6.3 | BIL(401) | IR75862(DP)/ Ce258 and ZGX1(RP) | Xu et al., 2015 |
|  | Fe | 11 | *qFe11* | RM441– RM202 | 4.5 | BIL(401) | IR75862(DP)/ Ce258 and ZGX1(RP) | Xu et al., 2015 |
|  | Zn | 6 | *qZn6* | RM3– RM340 | 24.8 | BIL(401) | IR75862(DP)/ Ce258 and ZGX1(RP) | Xu et al., 2015 |
|  | Zn | 7 | *qZn7* | RM134– RM1132 | 2.0 | BIL(401) | IR75862(DP)/ Ce258 and ZGX1(RP) | Xu et al., 2015 |
|  | Zn | 3 | *qZn3-1* | ad03013905-ad03014175 | 18.0 | DH (123lines) | 93-11/Milyang 352 | Lee et al., 2020 |
|  | Fe | 3 | *qFe3-1* | ad03014175- KJ03_069 | 10.7 | DH (123 lines) | 93-11/Milyang 352 | Lee et al., 2020 |
|  | Fe | 9 | *qFe9.1* | 9809545–9819278 | 11.79 | DH(148 lines) | IR05F102/IR69428 | Calayugan et al., 2020 |
|  | Fe | 12 | *qFe12.1* | 12702072–12732307 | 13.34 | DH(148 lines) | IR05F102/IR69428 | Calayugan et al., 2020 |
|  | Zn | 1 | *qZn1.1* | id1008679–439764 | 8.96 | DH(148 lines) | IR05F102/IR69428 | Calayugan et al., 2020 |
|  | Zn | 5 | *qZn5.1* | 4904312–4908650 | 12.15 | DH(148 lines) | IR05F102/IR69428 | Calayugan et al., 2020 |
|  | Zn | 9 | *qZn9.1* | 9809545–9819278 | 13.79 | DH(148 lines) | IR05F102/IR69428 | Calayugan et al., 2020 |
|  | Zn | 12 | *qZn12.1* | c12p4887439–12172332 | 15.26 | DH(148 lines) | IR05F102/IR69428 | Calayugan et al., 2020 |
|  | Zn | 2 | *qZn2.1* | 2048774-2054640 | 12.2 | DH (pop1- 111 lines& pop2-146 lines) | IR64/IR69428 (Pop1)  BR29/IR75862 (Pop2) | Descalsota-Empleo et al., 2019 |
|  | Zn | 3 | *qZn3.1* | 3538410-3548096 | 12.2 | DH (pop1- 111 lines& pop2-146 lines) | IR64/IR69428 (Pop1)  BR29/IR75862 (Pop2) | Descalsota-Empleo et al., 2019 |
|  | Zn | 3 | *qZn3.1* | 3522453- 3538410 | 10.9 | DH (pop1- 111 pop2-146 lines) | IR64/IR69428 (Pop1)  BR29/IR75862 (Pop2) | Descalsota-Empleo et al., 2019 |
|  | Zn | 5 | *qZn5.1* | 5027770- 5077125 | 18.4 | DH (pop1- 111 & pop2-146 lines) | IR64/IR69428 (Pop1)  BR29/IR75862 (Pop2) | Descalsota-Empleo et al., 2019 |
|  | Zn | 5 | *qZn5.2* | 5645339- 5648872 | 11.5 | DH (pop1- 111 & pop2-146 lines) | IR64/IR69428 (Pop1)  BR29/IR75862 (Pop2) | Descalsota-Empleo et al., 2019 |
|  | Zn | 7 | *qZn7.1* | 7062019- 7089136 | 12.6 | DH (pop1- 111 lines& pop2-146 lines) | IR64 /IR69428 (Pop1)  BR29/IR75862 (Pop2) | Descalsota-Empleo et al., 2019 |
|  | Zn | 8 | *qZn8.1* | 8910035 8918570 | 8.6 | DH (pop1- 111 lines& pop2-146 lines) | IR64/IR69428 (Pop1)  BR29/IR75862 (Pop2) | Descalsota-Empleo et al., 2019 |
|  | Zn | 8 | *qZn8.1* | 8886338- 8904874 | 8.6 | DH (pop1- 111 lines& pop2-146 lines) | IR64/IR69428 (Pop1)  BR29/IR75862 (Pop2) | Descalsota-Empleo et al., 2019 |
|  | Zn | 9 | *qZn9.1* | wd9002310- 9831169 | 10.3 | DH (pop1- 111 lines& pop2-146 lines) | IR64/IR69428 (Pop1)  BR29/IR75862 (Pop2) | Descalsota-Empleo et al., 2019 |
|  | Zn | 11 | *qZn11.1* | 10907196- id11001107 | 27.7 | DH (pop1- 111 lines& pop2-146 lines) | IR64/IR69428 (Pop1)  BR29/IR75862 (Pop2) | Descalsota-Empleo et al., 2019 |
|  | Fe | 4 | *qFe4.1* | 4733006- 4743351 | 9.4 | DH (pop1- 130 & pop2- 97 lines) | PSBRc82 / Joryeongbyeo(P1) and PSBRc82 /IR69428 (P2). | Swamy et al., 2018 |
|  | Zn | 2 | *qZn2.1* | 2110566 id2009463 | 17.3 | DH (pop1- 130 & pop2- 97 lines) | PSBRc82 /Joryeongbyeo (P1) and PSBRc82/ IR69428 (P2). | Swamy et al., 2018 |
|  | Zn | 2 | *qZn2.1* | 2140834- 2147095 | 10.3 | DH (pop1- 130 & pop2- 97 lines) | PSBRc82/Joryeongbyeo (P1) and PSBRc82 / IR69428 (P2). | Swamy et al., 2018 |
|  | Zn | 3 | *qZn3.1* | 2783884-2785595 | 20.3 | DH (pop1- 130 & pop2- 97 lines) | PSBRc82/Joryeongbyeo (P1) and PSBRc82/IR69428 (P2). | Swamy et al., 2018 |
|  | Zn | 6 | *qZn6.1* | 6025827- 6047367 | 15.3 | DH (pop1- 130 & pop2- 97 lines) | PSBRc82 /Joryeongbyeo (P1) and PSBRc82 x IR69428 (P2). | Swamy et al., 2018 |
|  | Zn | 6 | *qZn6.2* | 6063412- id6006214 | 16.1 | DH (pop1- 130 & pop2- 97 lines) | PSBRc82/Joryeongbyeo (P1) and PSBRc82 x IR69428 (P2). | Swamy et al., 2018 |
|  | Zn | 8 | *qZn8.1* | 8803052- 8832534 | 14.1 | DH (pop1- 130 & pop2- 97 lines) | PSBRc82 /Joryeongbyeo (P1) and PSBRc82 x IR69428 (P2). | Swamy et al., 2018 |
|  | Zn | 11 | *qZn11.1* | 10858811 id11000778 | 22.8 | DH (pop1- 130 & pop2- 97 lines) | PSBRc82/x Joryeongbyeo (P1) and PSBRc82 x IR69428 (P2). | Swamy et al., 2018 |
|  | Zn | 12 | *qZn12.1* | id12008557 12985052 | 7.5 | DH (pop1- 130 & pop2- 97 lines) | PSBRc82/x Joryeongbyeo (P1) and PSBRc82 x IR69428 (P2). | Swamy et al., 2018 |
|  | Zn | 12 | *qZn12.2* | 13048465 13057679 | 12.2 | DH (pop1- 130 & pop2- 97 lines) | PSBRc82 /Joryeongbyeo (P1) and PSBRc82 x IR69428 (P2). | Swamy et al., 2018 |
|  | ZPR | 2 | *qZPR 2.1* | RM1367-RM262 | 11.3 | 190 RILs | PR116/Ranbir Basmati | Suman et al., 2021 |
|  | ZPR | 9 | qAOC_ZPR.9.1 | RM160–RM23669 | 6.23 | 190 RILs | PR116 /Ranbir Basmati | Suman et al., 2021 |
|  | IPR | 9 | qAOC_IPR.9.1 | RM6543–RM296 | 7.52 | 190 RILs | PR116 /Ranbir Basmati | Suman et al., 2021 |
|  | IBR | 1 | qIBR.1.1 | RM11741-RM11740 | 6.48 | 190 RILs | PR116 /Ranbir Basmati | Suman et al., 2021 |
|  | IBR | 1 | qAOC_IBR.1.1 | RM11741–RM11740 | 6.26 | 190 RILs | PR116 /Ranbir Basmati | Suman et al., 2021 |
|  | IBR | 5 | qIBR.5.1 | RM18904–RM18799 | 10.11 | 190 RILs | PR116 /Ranbir Basmati | Suman et al., 2021 |
|  | IBR | 5 | qAOC_IBR.5.1 | RM18904–RM18799 | 11.74 | 190 RILs | PR116 /Ranbir Basmati | Suman et al., 2021 |
|  | ZPR | 1 | qZPR.1.1 | SNP_21667551–SNP_20715764 | 37.84 | 44 RILS | PR116 /Ranbir Basmati | Suman et al., 2021 |
|  | ZPR | 1 | qAOC_ZPR.1.1 | SNP_21667551–SNP_20715764 | 37.84 | 44 RILS | PR116 /Ranbir Basmati | Suman et al., 2021 |
|  | ZPR | 11 | qZPR.11.1 | SNP_27183634–SNP_24162931 | 15.47 | 44 RILS | PR116 /Ranbir Basmati | Suman et al., 2021 |
|  | ZPR | 11 | qAOC_ZPR.11.1 | SNP_27183634–SNP_24162931 | 15.47 | 44 RILS | PR116 /Ranbir Basmati | Suman et al., 2021 |
|  | ZBR | 1 | qZBR.1.1 | SNP 21667551-SNP 20715764 | 30.61 | 44 RILS | PR116 /Ranbir Basmati | Suman et al., 2021 |
|  | ZBR | 2 | qZBR.2.1 | SNP_21560813–SNP_21617658 | 19.84 | 44 RILS | PR116 /Ranbir Basmati | Suman et al., 2021 |
|  | IPR | 3 | qIPR.3.1 | SNP_21240772–SNP_21185917 | 34.76 | 44 RILS | PR116 /Ranbir Basmati | Suman et al., 2021 |
|  | IPR | 6 | qIPR.6.1 | SNP_29657204–SNP_7127152 | 15.3 | 44 RILS | PR116 /Ranbir Basmati | Suman et al., 2021 |
|  | IPR | 7 | qIPR.7.1 | SNP_16328271–SNP_15892815 | 12.67 | 44 RILS | PR116 /Ranbir Basmati | Suman et al., 2021 |
|  | IPR | 7 | qAOC_IPR.7.2 | SNP_28458370–SNP_28114223 | 15.62 | 44 RILS | PR116 /Ranbir Basmati | Suman et al., 2021 |
|  | IPR | 11 | qAOC IPR11.1 | SNP_4832736-SNP_22702777 | 31.65 | 44 RILS | PR116 /Ranbir Basmati | Suman et al., 2021 |
|  | IBR | 5 | qIBR.5.1 | SNP_24090722–SNP_24120920 | 33.03 | 44 RILS | PR116 /Ranbir Basmati | Suman et al., 2021 |
|  | IBR | 7 | qAOC_IBR.7.1 | SNP_15070854–SNP_16328271 | 22.13 | 44 RILS | PR116 /Ranbir Basmati | Suman et al., 2021 |
|  | Fe | 2 | *qFe2* | RM555 | 42.12 | 212 F5 RILs | PAU 201/ Palman 579 | Pippal et al., 2021 |
|  | Fe | 12 | *qFe12* | RM12 | 4.30 | 212 F5 RILs | PAU 201/ Palman 579 | Pippal et al., 2021 |
|  | Fe | 2 | *qFe2* | RM263.2 | 52.80 | 198 F6 RILs | PAU 201/ Palman 579 | Pippal et al., 2021 |
|  | Fe | 12 | *qFe12* | RM327 | 44.80 | 198 F6 RILs | PAU 201/ Palman 579 | Pippal et al., 2021 |
|  | Zn | 2 | *qZn2.1* | RM1092 | 14.59 | 212 F5 RILs | PAU 201/ Palman 579 | Pippal et al., 2021 |
|  | Zn | 2 | *qZn2.2* | RM406 | 7.31 | 212 F5 RILs | PAU 201/ Palman 579 | Pippal et al., 2021 |
|  | Zn | 2 | *qZn2.1* | RM555 | 64.6 | 198 F6 RILs | PAU 201/ Palman 579 | Pippal et al., 2021 |
|  | Zn | 2 | *qZn2.2* | RM263.2 | 44.4 | 198 F6 RILs | PAU 201/ Palman 579 | Pippal et al., 2021 |
|  | Zn | 6 | *qZn6* | RM162 | 2.60 | 198 F6 RILs | PAU 201/ Palman 579 | Pippal et al., 2021 |
|  | Zn | 10 | *qZn10* | RM474 | 45.70 | 198 F6 RILs | PAU 201/ Palman 579 | Pippal et al., 2021 |
|  | Zn | 12 | *qZn12.1* | RM1080 | 3.90 | 198 F6 RILs | PAU 201/ Palman 579 | Pippal et al., 2021 |
|  | Zn | 12 | *qZn12.2* | RM2734 | 28.4 | 198 F6 RILs | PAU 201/ Palman 579 | Pippal et al., 2021 |

**Source:** Adopted and modified from Mahender et al., (2016) & Sharma et al., (2020)
